# Microglial clearance, neuroprotection and cognitive recovery via a novel synthetic sulfolipid in Alzheimer’s disease

**DOI:** 10.1186/s12974-025-03634-w

**Published:** 2025-12-13

**Authors:** Carmela Gallo, Lucia Verrillo, Emiliano Manzo, Emma Cauzzi, Livia La Barbera, Giuseppina Sabatino, Maria Luisa De Paolis, Michele Francesco Maria Sciacca, Giusi Barra, Elisa Peroni, Olivier Monasson, Mario Dell’Isola, Dalila Carbone, Annalisa Nobili, Marcello Ziaco, Laura Fioretto, Marinella Pirozzi, Giuliana D’Ippolito, Daniela Castiglia, Andrea Soluri, Genoveffa Nuzzo, Danilo Milardi, Maria Giuseppina Miano, Marcello D’Amelio, Angelo Fontana

**Affiliations:** 1 CNR - Institute of Biomolecular Chemistry, Pozzuoli, Italy; 2 CNR - Institute of Genetics and Biophysics “Adriano Buzzati-Traverso” , Via Pietro Castellino 111, Neaples, 80131 Italy; 3 Department of Experimental Neurosciences, IRCCS Santa Lucia Foundation, Via del Fosso di Fiorano, 64 , Rome, 00143 Italy; 4 Department of Medicine and Surgery, Università Campus Bio-Medico di Roma, Via Alvaro del Portillo, 21, Rome, 00128 Italy; 5 CNR - Institute of Crystallography, Catania, Italy; 6 CY Cergy Paris Universite, CNRS, BioCIS UMR 8076, Cergy Pontoise, 95000 France; 7 Universite Paris-Saclay, CNRS, BioCIS UMR 8076, Orsay, 91400 France; 8 Dept. Biology of University of Naples “Federico II”, Neaples, Italy; 9 Dept. Chemical Sciences of University of Naples “Federico II”, Neaples, Italy; 10 CNR- Institute of Endotypes in Oncology, metabolism and Immunology “G.Salvatore”, Neaples, Italy; 11 Department of Medical and Surgical Sciences, Magna Graecia University, Catanzaro, Italy; 12 CNR, Institute of Biomolecular Chemistry, Catania, Italy

**Keywords:** Microglia, Alzheimer’s disease, Small molecule, TREM2, Amyloid-β, Neuroinflammation, Drug discovery, Dopamine neurons, Natural Product

## Abstract

**Background:**

Microglial dysfunction has emerged as a critical factor in the Alzheimer’s Disease (AD), with innate immune dysregulation driving pathological progression. This growing understanding has shifted attention toward the therapeutic modulation of glial response as a means to restore immune balance and promote neuronal resilience. Increasing attention has also focused on small-molecules capable of reprogramming microglia toward a reparative, surveillance-competent phenotype that enhances Amyloid-β (Aβ) clearance while limiting detrimental neuroinflammation. In this context, we previously reported that Sulfavant A (SULF A), a novel synthetic sulfolipid, binds to Triggering Receptor Expressed on Myeloid Cells 2 (TREM2) and activates myeloid cells without inducing inflammatory hallmarks.

**Methods:**

The immunomodulatory properties of SULF A were characterized using biochemical, molecular, and cell biological approaches in primary murine microglia. The small modulatory molecule SULF A was synthetically produced in our laboratory. Functional assays included quantitative analyses of phagocytosis using standard beads and fluorescently labeled Aβ peptides, designed and synthesized considering tag emission and fiber aggregation. Aβ aggregation was characterized using biophysical and biochemical methods to ensure well-defined assemblies in microglial assays. To assess translational relevance, SULF A was evaluated at two distinct time points, before and after Aβ plaque deposition, in Tg2576 transgenic mice, a well-established AD model, to determine its neuroprotective potential and therapeutic efficacy.

**Results:**

We demonstrate that the small-molecule TREM2 ligand SULF A functions as an immunomodulator that reprograms microglia toward a reparative phenotype. It promotes a ramified and polarized microglial morphology associated with enhanced cellular motility, phagocytic capacity and clearance of neurotoxic Aβ peptides. SULF A upregulates arginase expression, downregulates inducible Nitric Oxide Synthase (iNOS), and redirects microglial metabolism toward tissue-protective functions. This phenotypic shift is characterized by increased TREM2 turnover and upregulation of CD68 and CCL2, without triggering classical inflammatory hallmarks. In vivo, SULF A enhances plaque‑associated microglial recruitment, reduces plaque burden, preserves midbrain dopaminergic neurons, and improves cognitive and motivational performance in Tg2576 mice.

**Conclusions:**

We propose that SULF A is a promising therapeutic small molecule that restores microglial homeostasis via an inflammation-sparing mechanism. It enhances amyloid clearance, preserves vulnerable neurons, and improves cognitive function in vivo. These findings support SULF A as a disease-modifying approach that extends beyond conventional plaque-targeting strategies in AD.

**Supplementary Information:**

The online version contains supplementary material available at 10.1186/s12974-025-03634-w.

## Background

Alzheimer’s Disease (AD), the leading cause of late-onset dementia, is a progressive, multifactorial neurodegenerative disorder characterized by progressive cognitive decline and a profound societal and economic impact. While its etiology is not fully understood, the neuropathological hallmarks include extracellular Amyloid-β (Aβ) plaque deposition, intracellular neurofibrillary tangles, and widespread synaptic and neuronal loss. Accumulating evidence implicates chronic neuroinflammation and dysregulation of innate immune pathways, particularly those involving microglia, as central contributors to disease progression [1].

Microglia, the resident immune cells of the brain, play essential roles in maintaining neural circuit integrity through synaptic pruning and remodeling processes that are crucial for learning and memory [2, 3]. These cells also safeguard the Central Nervous System (CNS) microenvironment and mediate the clearance of apoptotic cells and protein aggregates through highly regulated phagocytic programs. Their activity is orchestrated *via* dynamic membrane protrusions and committed receptors, such as those responsive to Damage- and Pathogen-Associated Molecular Patterns (DAMPs and PAMPs) [4].

In the AD context, microglia initially exert neuroprotective functions by promoting Aβ clearance and supporting tissue repair [5]. Upon activation, they traverse a spectrum of functional states broadly characterized by pro-inflammatory and anti-inflammatory phenotypes, associated with initiation and resolution of neuroinflammation, respectively [6, 7]. As disease pathology intensifies, microglial function often becomes chronically dysregulated, favoring a persistent pro-inflammatory phenotype characterized by reduced phagocytic competence and increased secretion of neurotoxic mediators [8]. This shift is associated with microglial clustering around Aβ plaques and altered expression of receptors such as Triggering Receptor Expressed on Myeloid Cells 2 (TREM2), which governs key aspects of metabolic regulation, proliferation, and motility [9, 10].

Current therapeutic strategies have increasingly prioritized the modulation of glial responses as a mean to restore immune homeostasis and support neuronal resilience. Monoclonal antibodies directed against Aβ species emerged as a promising approach to reduce plaque burden and to slow cognitive decline, potentially exerting their effects, in part, through the modulation of glial dynamics [11, 12]. In parallel, there is growing interest in small-molecule approaches aimed at reprogramming microglia toward a reparative, surveillance-competent phenotype that enhances Aβ clearance and mitigates deleterious neuroinflammation [13, 14]. Among these, the cromolyn-ibuprofen combination ALZT-OP1 is currently undergoing clinical evaluation for its potential dual efficacy in reducing both amyloid pathology and neuroinflammatory burden [15].

Our recent investigations identified Sulfavant A (SULF A), a synthetic analog of natural sulfolipids originally developed as a vaccine adjuvant [16, 17], as a novel modulator of innate immunity with potential relevance in AD. Importantly, SULF A crosses the blood-brain barrier [18], enabling a possible direct modulation of CNS immune responses. Unlike traditional pattern recognition receptor agonists, SULF A engages TREM2 and elicits a context-dependent immune response favoring resolution over inflammation. It induces a regulatory dendritic cell phenotype (homeDCs) characterized by enhanced expression of antigen-presenting and co-stimulatory molecules with minimal pro-inflammatory cytokine production [19]. Importantly, its modulatory effects extend to the adaptive immune compartment, promoting regulatory T cell polarization and antigen-specific antibody responses [20].

Despite the absence of prior studies evaluating SULF A in AD context, its unique mechanism of action makes it an attractive candidate for microglial reprogramming to enhance Aβ clearance while avoiding chronic neuroinflammation. In the present study, we investigated the potential of SULF A to rewire microglial function toward a neuroprotective, homeostatic phenotype. To test the hypothesis that SULF A selectively enhances microglial immunosurveillance and Aβ phagocytic clearance, while concurrently attenuating neuroinflammatory cytotoxicity, we employed a multimodal experimental design, that includes both in vitro mechanistic assays and two age-points of a validated mouse model of AD. The primary objective was to elucidate the disease-modifying potential of SULF A as a small-molecule therapeutic agent capable of exerting sustained immunomodulatory and neuroprotective benefits across both prodromal and advanced neuropathological stages of AD.

## Materials and methods

### Animal welfare and ethical compliance

All animal procedures were performed in compliance with the European Community Directive 2010/63/EU and approved by the Italian Ministry of Health (D.Lgs. n. 26/2014), in accordance with the Institutional Animal Care guidelines of the Institute of Genetics and Biophysics “Adriano Buzzati-Traverso” (authorization numbers 307/2018-PR and 0009895-P). Experiments involving Tg2576 mice were conducted in compliance with ARRIVE guidelines and the ethical standards of the European Council Directive 2010/63/EU, with approval from the Italian Ministry of Health (#842/2019-PR).

### Animals

C57BL/6 mice were obtained from Charles River Laboratories (Wilmington, MA, USA). Pregnancy was confirmed *via* detection of a vaginal plug, designated as embryonic day 0.5 (E0.5). Newborn pups (P0–P1) were sacrificed by decapitation. Brains were rapidly dissected under a stereomicroscope in ice-cold PBS.

Heterozygous Tg2576 mice (Taconic, APPSWE Model 1349) from 45 day-old to 6 months of age and at 12–13 months of age were used. Mice were housed under a 12 h light/dark cycle with *ad libitum* access to food and water. Environmental enrichment was standardized across cages (2–3 animals per cage).

### SULF A preparation

SULF A was chemically synthesized as described by Ziaco et al. [21]. Compound purity was determined by mass spectrometry and the product was tested on TLR2 and TLR4 assay to exclude the presence of TLR-active contaminants prior to use.

### Drug administration

For the longer treatment 45-day-old mice received intraperitoneal injections (i.p.) of SULF A (100 mg/kg in 0.1 M phosphate buffer saline, PBS) once a week until they reached 6 months of age. For the shorter treatment on aged mice, 12-month-old mice received the same i.p of SULF A twice a week for four weeks. Control animals received PBS alone. Tissues were collected one hour after the final dose.

### Primary murine microglial cultures and treatment

Primary murine microglia were isolated from neonatal (P0–P1) C57BL/6 mice. Whole brains, excluding cerebella, were enzymatically digested in serum-free DMEM containing 1.5 mg/mL trypsin and 0.1 mg/mL DNase I at 37 °C for 20 min. Cells were resuspended in DMEM supplemented with 10% fetal bovine serum (FBS), 1% penicillin/streptomycin, 2 mM L-glutamine, and 1 mM sodium pyruvate. Cells were cultured in T75 flasks for 14–15 days at 37 °C in a humidified incubator. Microglia were collected by mechanical agitation and purified using CD11b + magnetic selection (Miltenyi Biotec). Cell purity was assessed by flow cytometry (> 95%) using CD11b-VioBlue and CD45-FITC staining.

Purified microglia were plated on poly-D-lysine–coated surfaces and treated with 10 µg/mL SULF A in PBS for 24 or 48 h.

### Flow cytometry

Surface markers were assessed by staining with anti-mouse CD11b-VioBlue, CD40-PE, CD86-FITC, CD200R-APC (Miltenyi Biotec), and TREM2-APC (R&D Systems) according to standard protocols. Dead cells were excluded using propidium iodide (1:1000, Thermo Fisher Scientific). For intracellular iNOS and ARG-1 detection, cells were fixed and permeabilized using the BD Cytofix/Cytoperm kit, then stained with iNOS-FITC and ARG-1-PE (Thermo Fisher Scientific).

Samples were analyzed on BD Accuri C6 or MACSQuant Analyzer 16 flow cytometers. Data were processed using FlowJo v9 (Tree Star) or BD Accuri software.

### RNA extraction and gene expression

Total RNA was isolated with TRIzol reagent per manufacturer’s instructions. RNA concentration and purity (A260/A280 ratio) were determined using a NanoDrop 2000 spectrophotometer. cDNA was synthesized using the SuperScript III Reverse Transcriptase kit (Life Technologies).

mRNA expression levels were quantified by real-time PCR using SYBR Green chemistry and primers listed in Supplementary Table 1. Reactions were performed in triplicate across three biological replicates.

### ELISA

Concentrations of IL-12p70, IL-10, TNF-α, IL-4, and CCL2 in culture supernatants were determined using commercial ELISA kits (Thermo Fisher Scientific), following the manufacturer’s protocols.

### Nitric oxide assay

Nitrite production was assessed using the Griess Reagent Kit (Thermo Fisher Scientific). After 24–48 h of SULF A treatment, culture media were analyzed in 96-well plates. Absorbance was read at 548 nm using a 4300 Chromate microplate reader (Awareness Technology).

### Neuron–microglia co-cultures

Primary cortical neurons were prepared from WT neonatal brains and co-cultured with purified microglia at a 1:2 neuron-to-microglia ratio on DIV11. Cultures were treated with SULF A (10 µg/mL) and monitored by time-lapse microscopy for 24 h.

### Time-lapse imaging

Live-cell imaging was performed on a Zeiss Observer Z1 epifluorescence microscope with environmental control. Images were acquired every 20 min for 24 h and processed with ZEN Blue software.

### Phagocytosis assays

IgG-FITC Beads: primary murine microglia (3 × 10⁵ cells/well) were seeded in 24-well plates and allowed to adhere for 2 h. Cells were then incubated with SULF A (10 µg/mL) and IgG-FITC–coated beads (1:100 v/v; Cayman Chemical) for 3 h. After incubation, cells were washed, stained with propidium iodide, and analyzed by flow cytometry.

Phagocytosis rate was calculated as: 

1 $$\left(\left(\%\mathrm{sample}-\%\mathrm{control}\;\left(\mathrm{beads}\;\mathrm{only}\right)\right)/\%\mathrm{control}\right)\times100$$

*Escherichia coli* Particles: pHrodo Red-labeled E. coli particles (Thermo Fisher Scientific) were suspended in PBS (1 mg/mL), sonicated at 30 °C for 10 min, and vortexed. Cells were incubated with 10 µg/mL of both SULF A and bioparticles for 3 h. Vitality was assessed with Zombie Aqua viability dye. Data acquisition and analysis were performed as described above.

### Peptide synthesis, labeling, and aggregation

Aβ₁₋₄₀ labeled with 5(6)-carboxyfluorescein (fAβ) was synthesized *via* microwave-assisted solid-phase peptide synthesis (MW-SPPS) using Liberty Blue (CEM Corporation), following the Fmoc/tBu strategy. Full synthesis, purification, and characterization details are available in the Supplementary Information.

For monomerization, peptides were dissolved in HFIP, lyophilized, and reconstituted in phosphate buffer (10 mM, 100 mM NaCl, pH 7.4). To minimize the interference of the fluorescent probe, a stock solution of Aβ aggregates was prepared by incubating 50 µM Aβ₁₋₄₀ with 2.5 µM Aβ at 37 °C with gentle shaking. Aggregation was monitored using Thioflavin T fluorescence on a Victor Nivo 3S plate reader.

### fAβ phagocytosis assay

Primary murine microglia were treated with SULF A (10 µg/mL) and 1 µM 20:1 Aβ_1-40_/fAβ solution for 1 h. Cells were washed, stained with propidium iodide, and analyzed by flow cytometry. For imaging, cells were fixed in 4% paraformaldehyde and imaged by confocal microscopy.

### Immunofluorescence and tissue staining

Immunofluorescence in cultured primary murine microglia and mouse brain tissue was performed as described in the Supplementary Information. Images were acquired using Zeiss LSM 700 or Nikon Eclipse Ti2 confocal microscopes and analyzed using Fiji/ImageJ.

### Stereology

Sections processed for immunofluorescence were used to obtain estimates of numbers of TH^+^ neurons or of Iba1^+^ cells in the VTA [22]. The boundaries of the areas used for counting were defined by TH staining, and area distinction was performed according to published guidelines and as described in the text. We applied an optical fractionator stereological design (bilateral count) using the Stereo Investigator System (MicroBrightField Europe e.K.). A stack of MAC 5000 controller modules (Ludl Electronic Products, Ltd) was interfaced with an Olympus BX50 microscope with a motorized stage and a HV-C20 Hitachi digital camera with a Pentium II PC workstation. A 3D optical fractionator counting probe (x, y, z dimension of 50 × 50 × 25 μm for neurons and 100 × 100 × 25 μm for glia cells) was applied. The brain area of interest was outlined using the 5x objective and neuronal cells were marked with a 100x oil-immersion objective or a 40x-objective for microglial cells. Neurons were considered TH^+^ if they showed cytoplasmatic immunoreactivity. The total neuron numbers were estimated according to the formula (Eq. 1):

2 $$\mathrm N=\mathrm{SQ}\times\left(1/\mathrm{ssf}\right)\times\left(1/\mathrm{asf}\right)\times\left(1/\mathrm{tsf}\right)$$

where SQ represents the number of neurons counted in all optically sampled fields of the area of interest, ssf is the section sampling fraction, asf is the area sampling fraction and tsf is the thickness sampling fraction.

### Morphological analysis

Microglia were imaged with a Zeiss Microscope Axio Imager KMAT with motorized stage and a camera connected to Neurolucida software (7.5v; MBF Bioscience) for quantitative 3D-analysis of the entire microglial cell during live visualization [23] Only non-overlapping cells with clear soma and branching were considered for the analysis. Soma area and perimeter were measured; Sholl analysis included counting the number of ramification intersections, nodes and endings, and ramification lengths at fixed distances from the soma in 10 μm-spaced concentric circles originating from the soma. Analysis was done with an 100x-oil objective. Nine representative cells/animal were analyzed randomly, and data were averaged for each mouse.

### Open Field Test (OFT) and Novel Object Recognition Test (NORT)

Behavioral tests were performed in a dimly-lit (25 lx) plexiglass open field arena (60 × 60 × 30 cm), with dark-grey walls and white floor. On the first day of the testing battery, mice were assessed in the OFT. Each mouse was placed in the same corner of the arena and allowed freely-explore it for 10 min, during which locomotor activity and rearing behaviors were recorded. Thereafter, we conducted the NOR test, consisting of habituation, training, and testing [24, 25]. During habituation, mice were familiarized with the empty arena for 10 min. 24 h later (training phase), mice were exposed for 10 min to two identical objects (white wooden spheres) placed in the arena center equidistant from each other, then returned to their home cages. Following 24 h (testing phase), one of the familiar objects was replaced by a novel one (a light-grey wooden cone) and mice were allowed to explore them for 10 min. In both the training and testing sessions, the exploration time was recorded, calculated as the duration time (sec) in which mice touched or climbed on an object or sniffed it at a distance of at least 2 cm. Objects were randomized and counterbalanced across groups to minimize potential side or object preference. All objects and the arena were cleaned with 5% ethanol between sessions and mice.

### Statistical analysis

Statistical analyses were performed using GraphPad Prism v10.0. One-way ANOVA (Dunn’s post hoc), two-way ANOVA (Tukey’s post hoc), and unpaired two-tailed Student’s *t*-tests were applied as appropriate. Data distribution was assessed for normality utilizing the Shapiro–Wilk, Kolmogorov-Smirnov or D’Agostino & Pearson tests. Comparisons between two experimental groups (e.g., Tg PBS *vs*. Tg SULF A) were performed using 2-tailed parametric tests (unpaired *t*-test or Welch’s t-test) for data meeting the assumptions of Gaussian distribution, and non-parametric Mann–Whitney test for data that did not meet normality criteria. For datasets involving paired measurements, such as the training and testing phases of the NOR test, paired *t*-tests were used for normally distributed data, or Wilcoxon matched-pairs signed rank tests otherwise. Analyses involving comparisons among more than two groups (e.g., genotype *vs.* treatment; distance from Aβ plaque *vs.* treatment) were performed using two-way ANOVA. Sholl analysis was analyzed by two-way Repeated-Measures (RM) ANOVA, using distance from soma as repeated values. Post-hoc tests were performed using Sidak’s or Tukey’s multiple comparison tests. If no significant interaction was observed between the independent variables, statistical comparisons were conducted using *t*-tests. *P* < 0.05 was considered statistically significant.

## Results

### Transcriptional profiling reveals a non-polarized activation signature in microglia exposed to SULF A

**Fig. 1:**
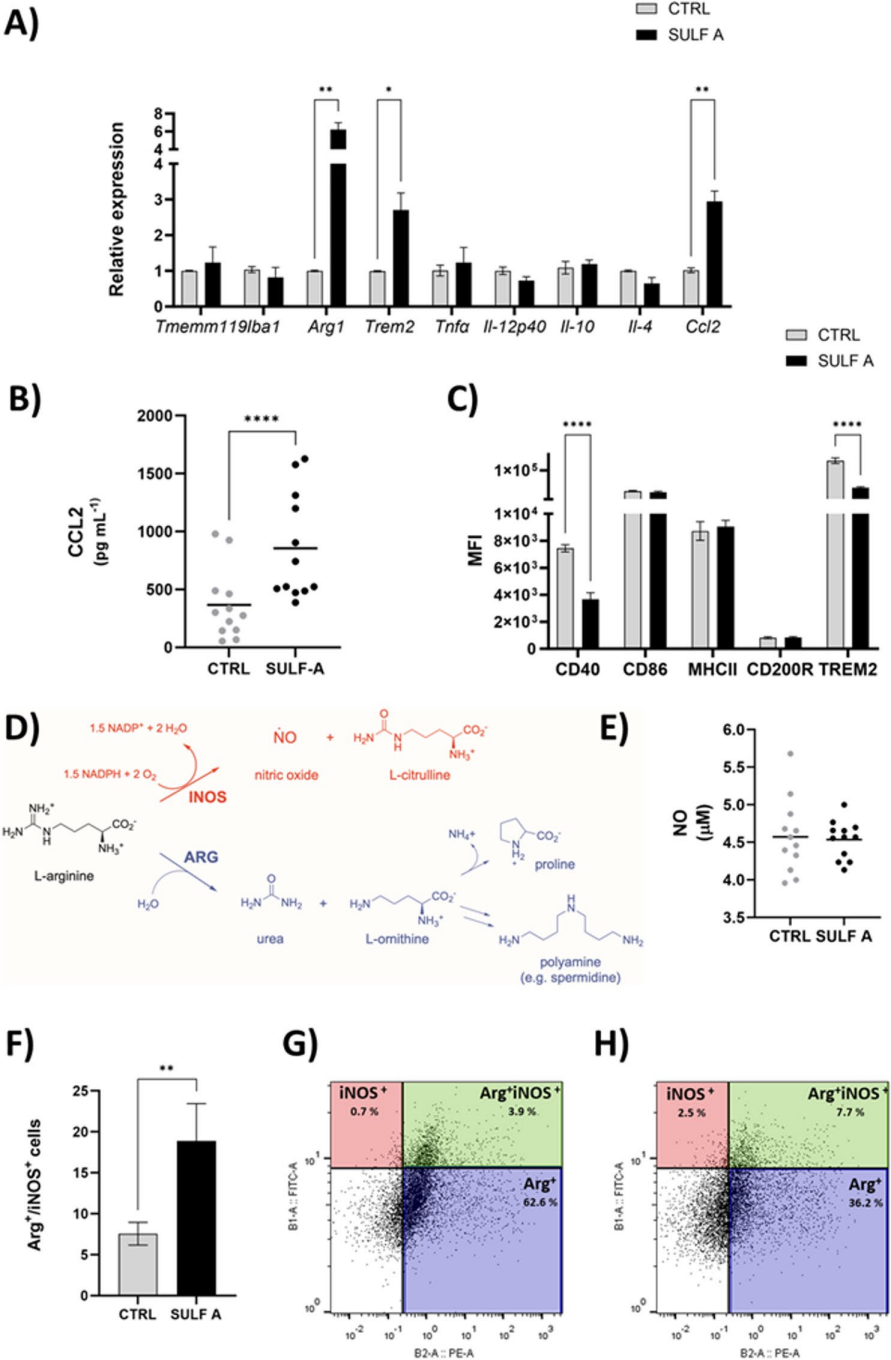
SULF A-dependent modulation of microglial activation and arginine metabolism. (**A**) RT-PCR analysis of cytokine and microglial marker gene expression following 24 h treatment. (**B**) CCL2 secretion quantified by ELISA at 48 h (n = 12). (**C**) Flow cytometric analysis of surface marker expression (CD40, CD86, MHC II, CD200R, TREM2) on untreated microglia (CTRL) or cells treated with 10 µg/mL SULF A for 24 h, expressed as mean fluorescence intensity (MFI; n = 6). (**D**) Schematic of iNOS/ARG metabolic pathways. (**E**) Quantification of nitric oxide (NO) release in culture supernatants by Griess assay at 48 h (n = 12). (**F**) Frequency of microglial cells expressing ARG, iNOS, or both, after 48 h of SULF A treatment (n = 6); (**G–H**) Representative FACS plots showing ARG⁺, iNOS⁺, and ARG⁺iNOS⁺ populations in untreated and SULF A-treated microglia. Data represent mean ± SEM. Statistical significance determined by paired t-test (P < 0.05; **P < 0.001; ***P < 0.0001)

To investigate the molecular response of primary murine microglia to SULF A, we first performed a transcriptional analysis following a 24-h stimulation with 10 µg/mL of the compound. Gene expression profiling revealed a marked upregulation of *Trem2*, Arginase 1 (*Arg1*) and chemokine (C-C motif) ligand 2 (*Ccl2*), whereas the expression of Transmembrane protein 119 (*Tmem119*) and Ionized calcium-Binding Adapter molecule 1 (*Iba1*), canonical markers of microglial identity and activation, remained unchanged (Fig. 1A). Likewise, transcripts encoding prototypical cytokines commonly used as indicators of pro-inflammatory (IL-12, TNF-α) and anti-inflammatory (IL-4, IL-10) phenotype were unaffected by SULF A treatment.

Although *Tmem119* and *Iba1* are frequently used to outline reactive or homeostatic microglial states, emerging evidence suggests their association with neuroinflammatory responses. Notably, reduced TMEM119 mRNA levels have been reported in AD, raising questions about its role in reactive microglia [26]. Their unchanged expression in this context suggests that SULF A does not engage classical pro-inflammatory activation pathways, in analogy with the response we reported in systemic DCs [19].

### Phenotypic and metabolic reprogramming suggests an Arg1-dominant, non-inflammatory microglial state

Complementing the transcriptional findings, secretome analysis revealed a selective increase in CCL2, while other inflammatory mediators remained unchanged or below the detection limit (Fig. 1B; Supplementary Information Fig. 1). The selective induction of CCL2, a chemokine involved in leukocyte recruitment and tissue homeostasis, further supports the emergence of a distinct transcriptional program, consistent with a non-polarized or context-dependent immune phenotype. These findings are consistent with previous observations in dendritic cells exposed to SULF A, where phenotypic maturation occurred in the absence of canonical inflammatory skewing [19]. In microglia, this transcriptional profile may reflect a homeostatic or adaptive response that modulates functional output without committing to a stereotypical M1/M2 polarization paradigm.

Flow cytometry analysis of surface markers demonstrated significant downregulation of CD40 and TREM2 following SULF A stimulation, with no changes observed in CD86, CD200R, or MHC class II expression (Fig. 1C). CD40, a member of the TNF receptor superfamily involved in co-stimulatory and inflammatory signaling, was particularly sensitive to SULF A, supporting the dampening of pro-inflammatory capacity [27, 28]. Notably, intracellular analysis revealed a paradoxical increase in TREM2 protein (data not shown) despite its membrane downregulation, suggesting ligand-induced receptor turnover or internalization in agreement with the response to targeting by specific antibodies [29]. Given the concurrent upregulation of *Trem2* transcripts, this redistribution likely represents a compensatory mechanism to maintain functional receptor availability. Such internal trafficking has been implicated in phagocytosis and exosome-mediated clearance of Aβ, underscoring a possible neuroprotective dimension to this response [30].

We next explored whether the observed increase in *Arg1* expression translated into functional reprogramming of arginine metabolism, which in immune cells, particularly macrophages and microglia, is controlled by ARG1 and the inducible Nitric Oxide Synthase (iNOS) [31]. The two enzymes are in competition for the substrate and convert *L*-arginine into *L*-ornhitine and Nitric Oxide (NO), respectively (Fig. 1D). Supernatant analysis revealed no detectable NO production up to 48 h post-stimulation (Fig. 1E). In contrast, intracellular ARG1 protein was robustly upregulated and iNOS levels were slightly reduced, resulting in a clear increase (4–5 fold) of ARG1/iNOS ratio relative to untreated controls (Fig. 1F). Moreover, SULF A induced the emergence of an ARG1^+^ microglial population, along with a distinct subset of CD11b^+^ cells co-expressing ARG1 and iNOS (Fig. 1G–H).

This metabolic shift reflects a redirection of *L*-arginine metabolism away from NO synthesis and toward the production of ornithine and polyamines, metabolites associated with tissue remodeling and wound repair [32]. Production of iNOS-induced NO or ARG-induced polyamines and prolines largely influences the pro- or anti-inflammatory functions of microglia [33]. Importantly, the absence of NO production combined with selective ARG1 induction indicates that SULF A promotes an ARG1-dominant phenotype, mitigating classical pro-inflammatory responses while supporting potentially reparative functions.

In analogy with the immune profile induced by SULF A in DCs [19], these findings delineate a unique microglial response to SULF A characterized by selective marker modulation, chemokine release and metabolic reprogramming, without evidence of full polarization toward pro- or anti-inflammatory states. This profile suggests that SULF A may engage regulatory or adaptive immune mechanisms with relevance to CNS homeostasis and repair.

### Microglia plasticity induced by SULF-A stimulation

**Fig. 2:**
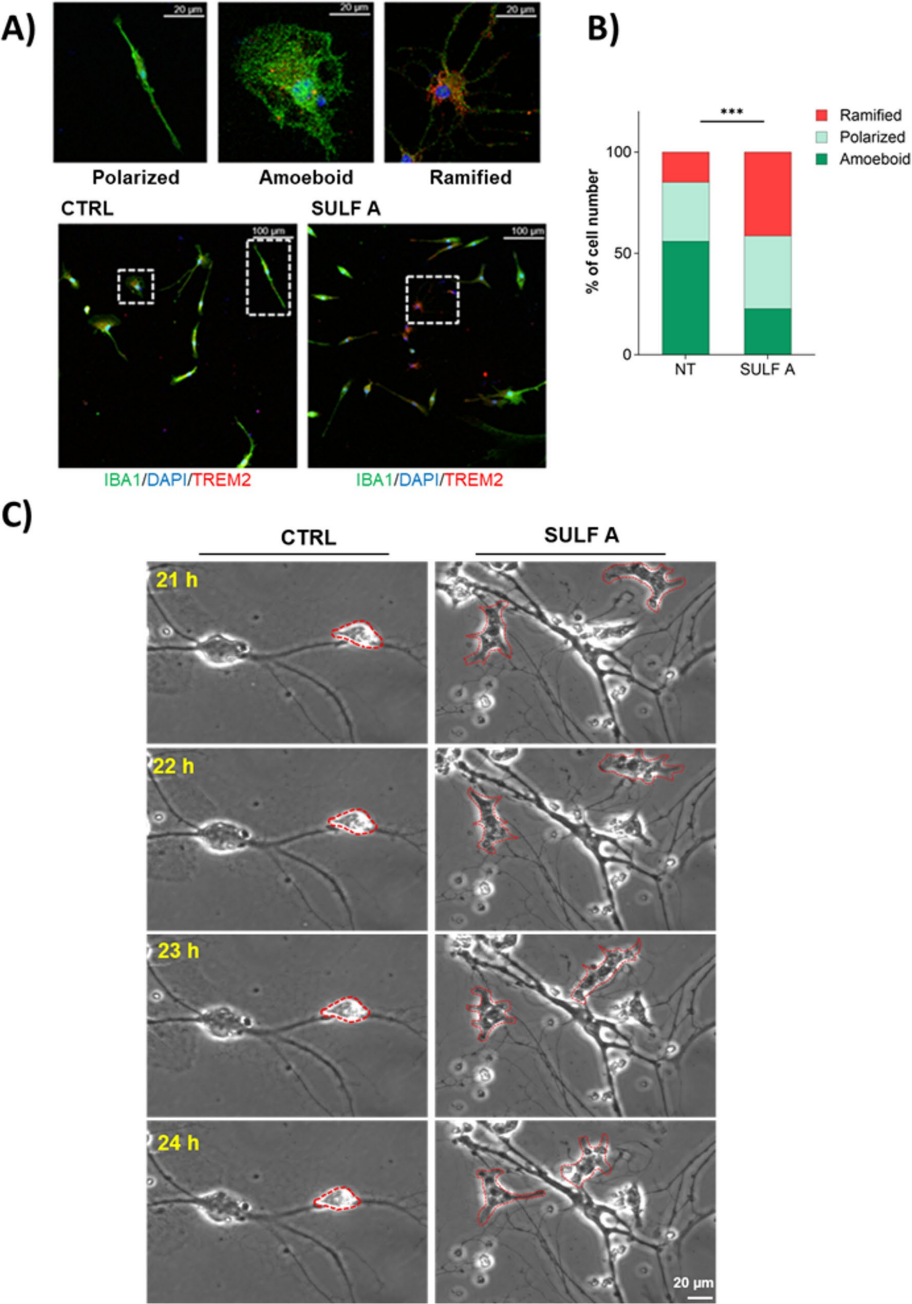
SULF A modulates microglial morphology and dynamics. (**A**) Representative confocal images showing double immunofluorescence staining for TREM2 (*red*) and Iba1 (*green*) in primary microglia. Nuclei were counterstained with DAPI (*blue*). Insets highlight distinct microglial morphologies—polarized, amoeboid, and ramified—at higher magnification. (**B**) Quantification of microglial morphology across at least 20 randomly selected fields per condition. A total of 796 cells (CTRL) and 754 cells (SULF A-treated) were analyzed. Statistical significance determined by Chi-square test; ***P < 0.0001. (**C**) Representative time-lapse images of primary microglia–neuron co-cultures acquired at 20-minute intervals over a 24-hour period. Red brackets indicate morphological changes in SULF A-treated microglia relative to untreated controls during the final 4 hours of imaging. Scale bar: 20 μm

Microglia are characterized by remarkable morphological plasticity, and their functional states are often categorized based on distinct morphological features. Under physiological conditions, these cells exhibit distinct morphologies, ranging from less activated rod-like (polarized) shapes to functional configurations with “amoeboid” or “ramified” forms [34, 35]. To assess the effects of SULF A on microglial morphology, primary mouse microglia were immunostained with Iba1 and TREM2 and classified in comparison to untreated controls (Fig. 2A). The analysis revealed a significant change in cell morphology of microglia treated with SULF A (Fig. 2B), resulting in the acquisition of a highly ramified morphology that is indicative of cells actively sensing the surrounding environment [36]. Along with the increase of ramified forms, we observed a decrease of phagocytic, amoeboid microglia displaying a swollen soma with fully retracted processes. It is interesting to note that these changes are not merely structural but are consistent with a possible TREM2 signaling, which has been implicated in cytoskeletal reorganization and motility [37, 38] TREM2 is a critical receptor in microglial biology. Together with the ability of SULF A to engage this receptor [19], the increased TREM2 transcription may underpin many of the observed functional changes [37, 39] and suggest a mechanistic basis for the effects of SULF A.

To support the emerging functions of SULF A, we also investigated the effect of the sulfolipid in co-culture of microglia with neurons by visual systems that are well-defined tools to investigate synapse plasticity. In time-lapse microscopy experiments (Fig. 2C), addition of SULF A revealed enhanced motility of microglial cells and frequent reshaping of their membranes with invaginations resembling phagocytic processes (Supplementary Movies 1–4). Concurrently, neurons showed increased branching compared to untreated controls, which could support a neurotrophic activity. Identification of neurotrophic small molecules that stimulate neuronal function, such as promoting neuritogenesis or neurite extension, strictly correlates with neurodegenerative disease research [40]. To the best of our knowledge, no sulfolipids or small molecules have been so far reported to exert neuroprotective and regenerative properties comparable to those of SULF A, nor have any small molecules showed these positive effects on the dynamic interaction between microglia and neurons. On the whole, the analysis of the effects of SULF A on morphology and motility supports the acquisition of a microglial phenotype with surveying and protective functions.

### Activation of microglial phagocytosis by SULF A

**Fig. 3:**
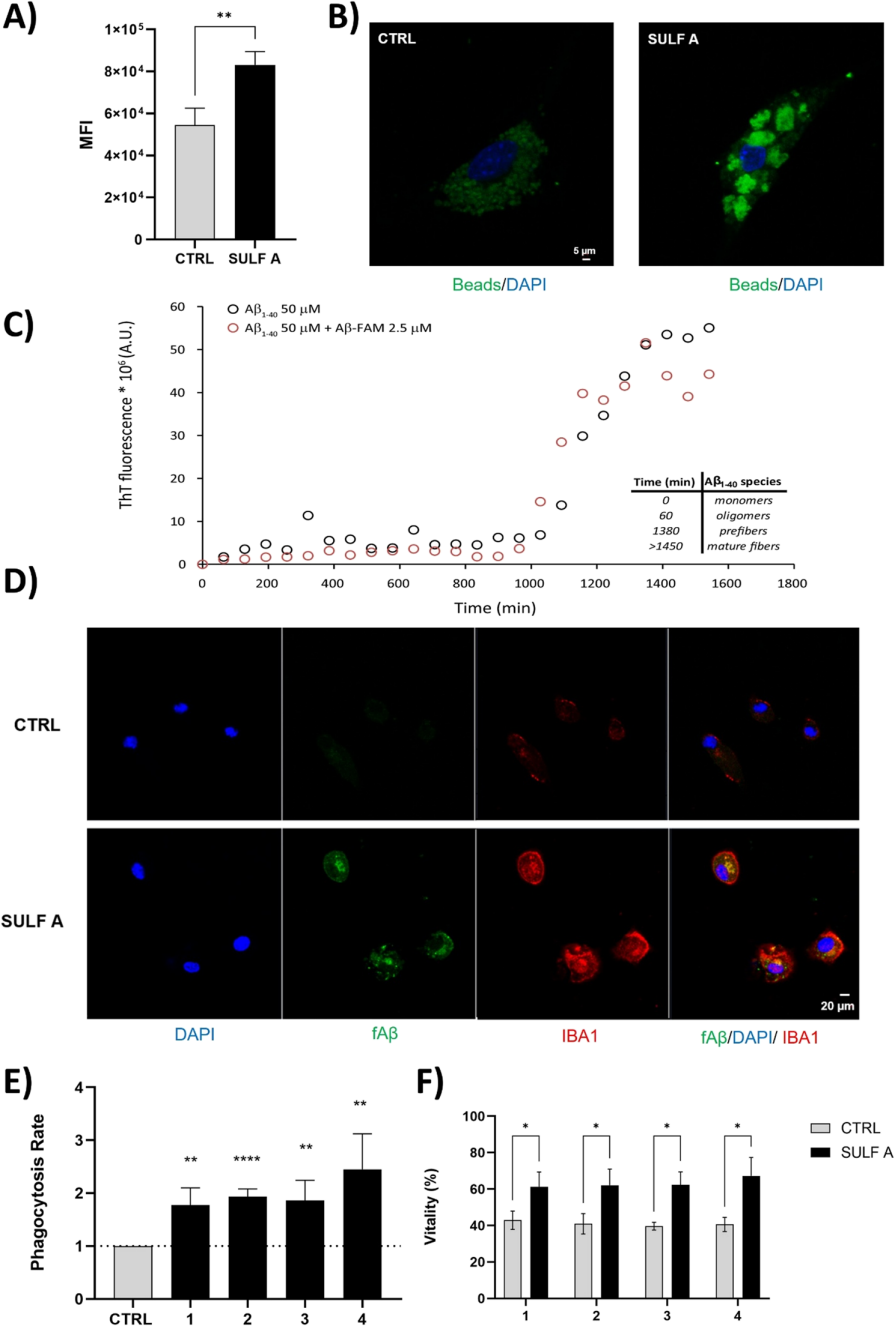
SULF A enhances microglial phagocytosis and protects against Aβ-induced cytotoxicity. (**A**) Quantification of latex bead uptake in neonatal microglia by flow cytometry after 3 h treatment with SULF A (10 µg/mL), expressed as MFI, (n = 6). **P < 0.01, by unpaired *t-*test. (**B**) Confocal images showing microglia nuclei stained with DAPI (*blue*) and phagocytosed FITC-labeled latex beads (*green*). Images acquired using a Zeiss LSM 700 confocal microscope with a 63×/NA 1.4 objective and zoom of 1.5–2×. Scale bar: 5 μm. (**C**) Thioflavin T fluorescence assay showing Aβ_1–40_ aggregation kinetics (50 µM Aβ_1–40_ alone, black; 50 µM Aβ_1–40_ + 2.5 µM fAβ_1–40_, *red*). Inset illustrates the time-dependent prevalence of Aβ species (monomers, oligomers, protofibrils, fibrils) at the time points of sample collection. (**D**) Representative confocal images of Iba1⁺ microglia (*red*) with internalized fibrillar fAβ (*green*) and DAPI (*blue*); CTRL = cells treated with only fAβ; SULF A = cells treated with fAβ and SULF A; Images acquired as in panel (B) Scale bar: 20 μm. (**E**) Phagocytosis assay conducted on primary murine microglia incubated for 3 h (n = 4) with monomers (1), oligomers (2), prefibers (3) and fibers (4) of fAβ; gray bar = untreated (CTRL); black bars = stimulated with 10 µg/mL SULF A; data are expressed as phagocytosis rate calculated from MFI after measurement of internalized fluorescence fibril intensity by flowcytometry (n = 3). (F) Analysis of microglial cell vitality following exposure to monomers (1), oligomers (2), prefibers (3) and fibers (4) of fAβ; data are expressed as percentage of live cells (%) in untreated cells (CTRL, gray bars) and cells stimulated with 10 µg/mL SULF A (black bars). Asterisks indicate significant differences from CTRL; ****P < 0.001; **P < 0.01; *P < 0.1 by unpaired t-test

Microglia, as professional phagocytes, play a critical role in maintaining brain homeostasis by clearing pathogens, apoptotic cells, extracellular protein aggregates, and superfluous synapses, thus preventing infection and modulating neuroimmune balance and synaptic functions [36, 41, 42]. To investigate whether SULF A modulates microglial phagocytosis, we initially employed flow cytometry to quantify uptake of fluorescently labelled IgG-coated latex beads. SULF A treatment significantly enhanced phagocytosis across six independent primary murine microglial cultures compared to untreated controls (Fig. 3A). This increase was further corroborated by confocal microscopy, which revealed augmented internalization of beads at single-cell level. Indeed, untreated microglia cells (CTRL) exhibited a diffuse and punctate bead distribution, whereas sulfolipid-treated microglia cells (SULF A) displayed prominent clusters of strongly internalized beads forming dense aggregate (Fig. 3B).

To elucidate the mechanism underlying this enhanced phagocytosis, microglia were incubated with non-opsonized *Escherichia coli* bioparticles (PhRodo Red). Notably, SULF A-treated cells did not exhibit increased uptake of these non-opsonic targets (Supplementary Information Fig. 2), indicating that the effect of SULF A may be dependent on opsonic receptor-mediated phagocytosis. Phagocytosis and phagocytic microglial phenotypes exist along a spectrum that is tightly regulated by diverse ligand-receptor interactions. The role of the complement system and opsonization has been extensively supported in recognition and engulfment of synapses [43] even if mitigation of the complement component C1q prevents excessive synapse loss and cognitive decline during aging [44]. Matteoli and co-workers reported correlation between microglia-mediated synaptic refinement and TREM2 levels in the AD context and neurodevelopmental diseases [45]. Notably, TREM2-dependent phagocytic mechanism can be mediated by CD36 [46], a class B scavenger receptor that can work synergistically to recognize and eliminate Low-Density-Lipoprotein (LDL)-bound group A *Streptococcus* (GAS) and lipid particles [47].

### SULF A-mediated microglial phagocytosis of Aβ aggregation forms

Microglial phagocytic activity is particularly critical for the innate immune response in the context of AD. The process plays a central role in the clearance of Aβ plaques [48]. Aβ peptides, mostly comprising 40 or 42 amino acid residues, are Intrinsically Disordered Proteins (IDPs) that undergo spontaneous self-assembly in solution to form soluble oligomers, protofibrils, and ultimately, highly ordered fibrils enriched in β-sheet structures [49]. These peptides originate from the sequential proteolytic cleavage of the amyloid precursor protein (APP) by β- and γ-secretases [50]. Accumulation of fibrillar Aβ species is clinically associated with exacerbation of neuroinflammatory responses and subsequent neuronal loss [51]. However, mounting evidence supports the hypothesis that small, low-organized Aβ oligomers represent the most toxic species in the amyloid cascade, implicating these early aggregation intermediates as key drivers of synaptic dysfunction and neurotoxicity [52, 53].

To investigate the dynamics of Aβ aggregation and its cellular uptake, we prepared fluorescently Aβ (fAβ) by N-terminal labelling of Aβ_1–40_ peptides with 5(6)-carboxyfluorescein (FAM) through microwave-assisted solid-phase synthesis. Aβ_1–40_ was selected due to its predominant occurrence over Aβ_1–42_ in brain tissue (9:1 molar ratio) [54]. As shown in Fig. 3C, FAM conjugation (red circle) did not affect the aggregation kinetics relative to the unlabeled peptide (black circle). Time-resolved sampling at 0, 1, 24, and 48 h yielded discrete fAβ species representing distinct aggregation states (Inset Fig. 3C). The effect of SULF A on the phagocytic uptake of amyloids was assessed using confocal microscopy (Fig. 3D; Supplementary Information Figs. 3, 4, 5 and 6) and quantified by flow cytometry, following incubation of primary murine microglia with 1 µM fAβ in monomeric, oligomeric, protofibrillar, and fibrillar forms (Fig. 3E).

Exposure of these cells to fAβ significantly induced a morphology change from a ramified to a rod-like or amoeboid shape (Supplementary Information Figs. 3D and 5), as previously observed [52, 53].

Notably, SULF A significantly enhanced the microglial morphological change and the uptake across all fAβ forms, including the less organized oligomeric species. In addition, SULF A conferred cytoprotective effects, increasing microglial viability and promoting a 50% recovery from fAβ-induced cytotoxicity (Fig. 3F). Altogether, these findings highlighted compelling capability of SULF A to potentiate microglial-mediated clearance of neurotoxic Aβ aggregates and to mitigate Aβ-driven cell damage.

### SULF A enhances microglial recruitment and phagocytic activity at advanced stages of Aβ pathology

To investigate the impact of SULF A on neuroimmune dynamics and amyloid pathology, we utilized Tg2576 mice, a widely used transgenic model of AD that overexpresses human APP with the Swedish mutation (K670N/M671L) which enhances β-secretase processing, leading to elevated production of Aβ peptides. These mice begin to exhibit amyloid plaque deposition from 10-11 months of age, accompanied by prominent microglial activation surrounding plaques throughout the neocortex and hippocampus from 10 to 16 months.

**Fig. 4:**
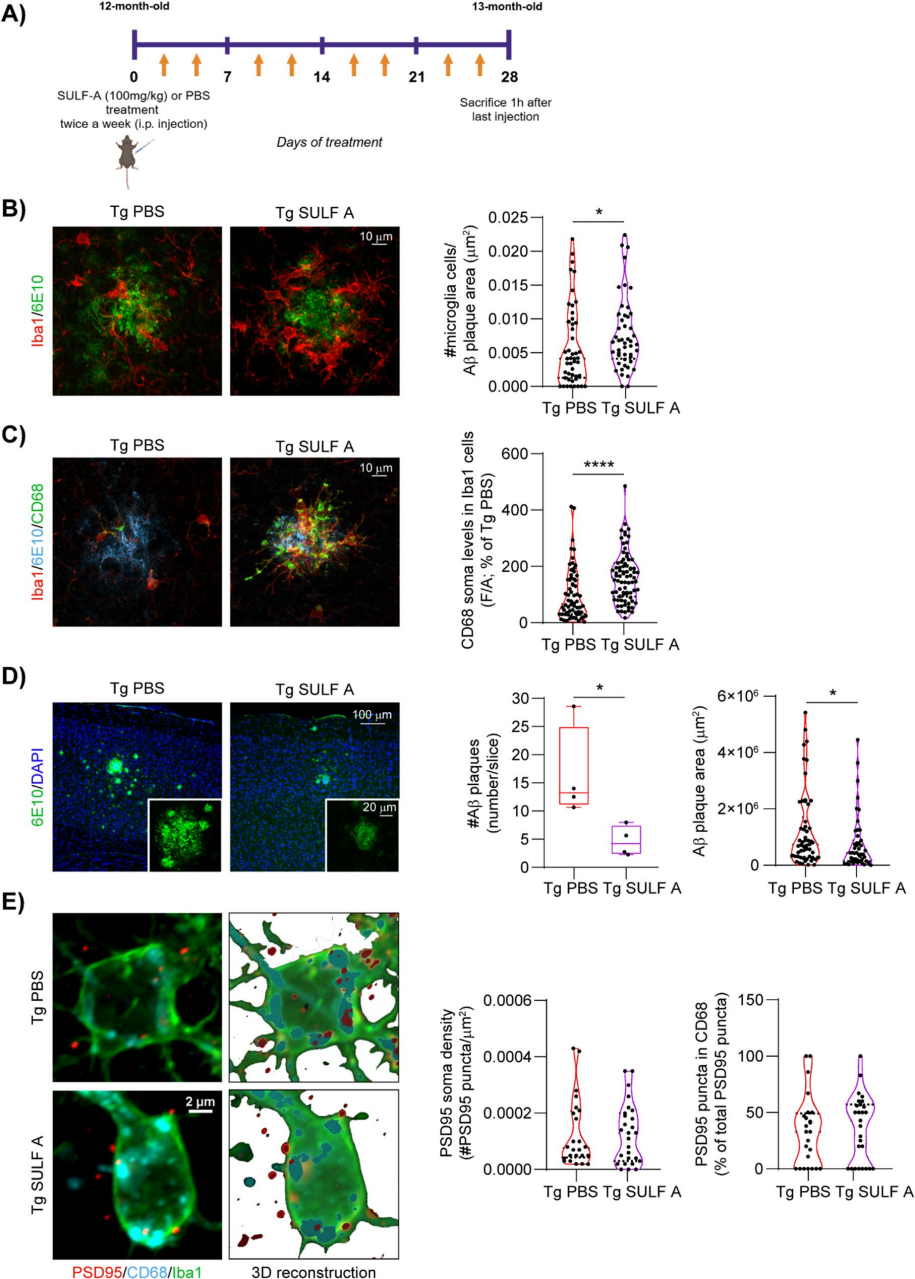
SULF A stimulates microglia to become phagocytic and infiltrate in Aβ plaques. (**A**) Schematic representation of *in vivo *treatment in aged mice. (**B**) Representative confocal images and plot showing the number of microglia (Iba1; *red*) within and around Aβ plaque (6E10, *green*; scale bar: 10 μm). Tg PBS and Tg SULF -A: n = 48 cells; n = 3 mice per group; With Mann-Whitney test: *P = 0.0216 (**C**) Representative confocal images of CD68 (*green*) in microglia cells (*red*) surrounding Aβ plaque (6E10, *blue*; scale bar: 10 μm). The plot shows CD68 somatic levels in microglia cells associated with Aβ plaque (Tg PBS: n = 67 cells/22 Aβ plaques; Tg SULF A: n = 78 cells/16 Aβ plaques; n = 3 mice per group; Mann-Whitney test: ****P < 0.0001). (**D**) Representative confocal images of Aβ plaque (6E10, *green*) in the cortex of Tg2576 mice treated with PBS or SULF A (scale bar: 100 μm). Nuclei are counterstained with DAPI. The insets (scale bar: 20 μm) show individual plaques. The plots show the number of Aβ plaque (*left*) and plaque area (*right*) (Plaque number: n = 4 mice per group; *P = 0.0345 unpaired *t-*test; Plaque area: Tg2576 PBS: n = 54 plaques; Tg2576 SULF A: n = 47 plaques; n = 4 mice per group; *P = 0.0179 Mann-Whitney test). (**E**) Representative confocal images of PSD95 puncta (*red*) within CD68 (*cyan*) in microglia cell (*green*) near Aβ plaque and relative 3D reconstructions (scale bar: 2 μm) in Tg2576 mice treated with PBS or SULF A. The plots show the PSD95 density on Iba1+ soma (*left*) and % of PSD95 puncta within CD68 in microglia cell associated with Aβ plaque (*right*; Tg PBS: n = 24 microglia; Tg SULF A: n = 27 microglia; n = 4 mice per group). [Schematics were created using BioRender (https://biorender.com)]

At 12-months of age, when widespread cortical and subcortical Aβ deposition is evident [55, 56], the animals received bi-weekly i.p. injections of SULF A or vehicle (PBS) for four weeks (Fig. 4A). Immunohistochemical analysis of cortical regions revealed a significant increase in Iba1 expressing (Iba1^+^) microglial density surrounding Aβ plaques in SULF A-treated mice compared to vehicle-treated Tg2576 controls, indicative of enhanced microglia recruitment to amyloid-laden regions (Fig. 4B). Functional activation was evaluated by quantifying CD68, a lysosomal marker of phagocytosis. CD68 immunoreactivity was higher in plaque-associated microglia than in cells distal to Aβ deposits (Supplementary Information Fig. 7A). In SULF A-treated mice, plaque-associated microglia showed further increases in CD68 compared with vehicle controls, consistent with enhanced phagolysosomal activity (Fig. 4C; Supplementary Information Fig. 7A). By contrast, CD68 in plaque-distant microglia was unchanged between SULF A and vehicle (Supplementary Information Fig. 7A).

To assess whether the SULF A-induced increase in plaque-associated microglia phagocytosis translates into reduced Aβ plaque burden, we quantified cortical Aβ plaque number and size in Tg2576 mice treated with either SULF A or vehicle. Consistent with the elevated phagocytic activity, SULF A-treated mice displayed fewer and smaller Aβ plaques compared to vehicle-treated controls (Fig. 4D). We next investigated whether the increase in microglial phagocytic activity extended beyond Aβ clearance to promote excessive synaptic pruning following prolonged SULF A treatment. To this end, we quantified immunolabeled PSD95^+^ puncta (post-synaptic dendritic spine marker) within CD68^+^ microglial lysosomes. Neither the density of PSD95^+^ puncta within microglia nor the proportion of PSD95^+^ puncta colocalized with CD68^+^ lysosomes differed between groups (Fig. 4E; Supplementary Information Fig. 7B). Collectively, these data support a model in which SULF A enhances microglial chemotaxis and phagocytic capacity to promote Aβ plaque surveillance and clearance without perturbing synaptic elements.

### SULF A preserves midbrain dopaminergic neurons and attenuates early behavioral deficits in an AD mouse model

**Fig. 5:**
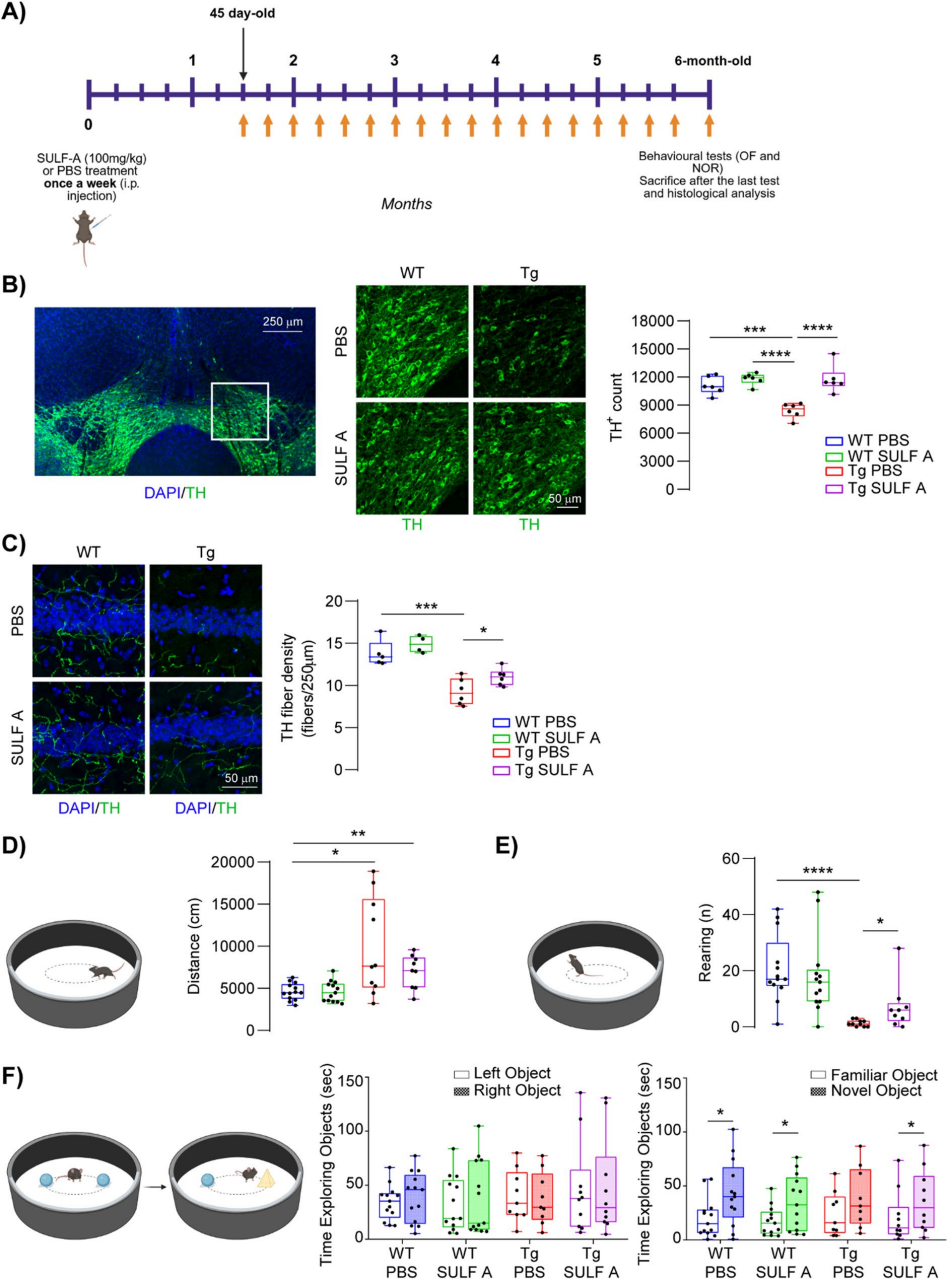
SULF A prevents VTA dopaminergic neuronal loss and behavioral deficits in 6-month-old Tg2576 mice. (**A**) Schematic representation of *in vivo* treatment in young mice. (**B**) Representative confocal images (TH, *green;* scale bar: 250 μm; magnification 50 μm) and plot showing stereological quantification of total TH^+^ cell numbers in the VTA of 6-month-old mice treated with PBS or SULF A (n = 6 per group; two-way ANOVA for Genotype *vs*. Treatment: Interaction: F (1, 20) = 10.43, p = 0.0042; Genotype: F (1, 20) = 10.98, p = 0.0035; Treatment: F (1, 20) = 24.01, P < 0.0001; WT PBS *vs.* Tg PBS: *** P = 0.0009; WT SULF A *vs.* Tg PBS: **** P < 0.0001; Tg PBS *vs*. Tg SULF A: **** P < 0.0001 with Tukey’s post-hoc test). (**C**) Confocal images and plots showing dopaminergic hippocampal fiber density (expressed as fibers/250 µm. WT PBS: n = 5; WT SULF A: n = 4; Tg PBS and Tg SULF A: n = 6 mice. Two-way ANOVA for Genotype *vs.* Treatment: Interaction F (1, 17)] = 0.2943, p = 0.5945. Unpaired *t*-test: WT PBS *vs*. Tg PBS: *** P = 0.001; Unpaired *t*-test: Tg PBS *vs*. Tg SULF A: * P = 0.046). Nuclei are counterstained with DAPI (scale bar: 50 μm). (**D**) The plot shows total locomotor activity, measured as the distance travelled in the OFT by WT and Tg2576 mice (WT PBS and WT SULF A: n = 13; Tg PBS: n = 10; Tg SULF A: n = 9 mice. Two- way ANOVA for Genotype *vs.* Treatment: Interaction: F (1, 41) = 2.558, P = 0.1174. Welch’s *t*-test: WT PBS *vs.* Tg PBS **** P < 0.0001; Mann-Whitney test: WT PBS *vs.* Tg SULF A ** P = 0.0046). (**E**) The plot shows the number of rearing attempts by mice in the OF arena (WT PBS and WT SULF A: n = 13; Tg PBS: n = 10; Tg SULF A: n = 9 mice. Two-way ANOVA for Genotype *vs*. Treatment: Interaction: F (1, 41) = 1.776, P = 0.1900. Welch’s *t-*test: WT PBS *vs.* Tg PBS: **** P < 0.0001; Mann-Whitney test: TG PBS *vs.* Tg SULF A: * P = 0.0281). (**F**) The plots show the exploration time spent by mice with the novel and/or familiar object during the NOR training (*left*) and test (right) session 24 h after training (WT PBS and WT SULF A: n = 13; Tg PBS: n = 9; Tg SULF A: n = 10 mice. Paired *t*-test: WT PBS * P = 0.0141; WT SULF A * P = 0.0407; WT PBS * P = 0.0141; Tg SULF A * P = 0.0405). [Schematics were created using BioRender (https://biorender.com)]

The ability of SULF A to target oligomeric and prefibrillar Aβ species underscores its potential for early intervention strategies aimed at preventing or delaying the progression of AD. To study the neuroprotective efficacy of SULF A during early AD pathogenesis, Tg2576 mice were administered weekly with i.p. injections SULF A or vehicle (PBS), starting at pre-symptomatic stage (postnatal day 45) and continuing through the pre-plaque symptomatic phase, up to 6 months of age. Mice were evaluated for neuronal integrity as well as cognitive and non-cognitive behavioral outcomes (Fig. 5A). At this stage, Tg2576 mice exhibit dopaminergic neuronal loss in the Ventral Tegmental Area (VTA) and impaired dopaminergic signaling in VTA-projection areas, such as the hippocampus, alterations previously shown to underlie deficits in hippocampal-dependent cognition [24, 57, 58].

Consistent with the earlier studies, stereological analysis confirmed a reduction in the number of Tyrosine Hydroxylase-positive (TH^+^) neurons in the dopaminergic VTA of PBS-treated Tg2576 mice compared to WT littermates. Interestingly, we found a striking preservation of VTA TH^+^ neurons in SULF A-treated Tg2576 mice, with cell numbers comparable to healthy controls and significantly higher than those observed in vehicle-treated transgenic Tg2576 mice (Fig. 5B). In addition, TH immunostaining revealed a pronounced loss of hippocampal dopaminergic fibers in Tg2576 mice compared to WT controls, an effect significantly mitigated by SULF A treatment (Fig. 5C). Notably, this SULF A-mediated neuroprotection appears to be independent of neuroinflammatory modulation, as no alterations in microglial cell number or morphology were detected between SULF A- and PBS-treated Tg2576 mice (Supplementary Information Fig. 8).

To determine whether the neuroanatomical preservation conferred by SULF A was translated into functional improvements, Tg2576 mice were subjected to behavioral tests probing VTA-hippocampus-dependent circuits. In the OFT, PBS-treated Tg2576 mice exhibit increased locomotion compared to WT controls, consistent with the hyperactive phenotype characteristic of this model [59–61]. While SULF A treatment did not alter overall locomotion, it significantly enhanced rearing behavior in Tg2576 mice compared to vehicle-treated transgenics (Fig. 5D-E), suggesting enhanced exploratory drive and improved dopaminergic tone [62]. In the NORT, SULF A-treated mice significantly improved object discrimination performance (Fig. 5F, right panel), indicating preserved recognition memory and a resistance to cognitive decline. Of note, exploration time during the training session was comparable across groups (Fig. 5F, left panel), ruling out potential confounds related to motivation state, sensorimotor capacity, or innate object preference. Collectively, these findings highlight that early and sustained SULF A administration preserves dopaminergic circuit integrity and ameliorates behavioral impairment associated with prodromal stages of AD pathology in Tg2576 mice.

## Discussion

Microglia play a dual role in the CNS, acting as both defenders and potential contributors to neuronal damage [63, 64]. Recent clinical breakthroughs in AD therapy have reignited debate about the role of Aβ in disease progression. While monoclonal antibodies such as lecanemab and donanemab demonstrate robust plaque clearance, they provide only moderate cognitive benefit and carry substantial risks, including Amyloid-Related Imaging Abnormalities (ARIA) and cerebral oedema [11, 65]. These limitations underscore the urgent need for alternative therapeutic strategies that go beyond plaque reduction, targeting immune dysfunction and synaptic resilience to deliver meaningful, durable neuroprotection.

Here, we show that SULF A, a synthetic immunomodulatory small molecule, promotes a neuroprotective microglial phenotype and provides therapeutic benefits in both early and late stages of experimental AD model. Indeed, in young Tg2576 mice, prior to overt plaque deposition, SULF A treatment preserved the structural integrity of dopaminergic circuits in the VTA and hippocampus, two regions vulnerable to early neurodegeneration in AD [66]. Behavioral testing in these animals showed improved recognition memory, indicating preservation of key cognitive circuits. This is consistent with emerging evidence that immune restoration may offer more clinically relevant benefit [67, 68]. Moreover, the increase in rearing behavior observed in SULF A-treated Tg2576 mice is particularly relevant, as this specific exploratory response has been shown to depend on VTA activity and its modulation by septal afferents, providing a functional readout of midbrain integrity [62]. In aged, plaque-bearing Tg2576 mice, SULF A significantly increased the peri-plaque accumulation of Iba1⁺ microglia and their CD68⁺ lysosomal compartments, indicating robust recruitment and phagocytic activation at amyloid deposits. This microglial engagement translated into a measurable reduction in cortical amyloid burden, with decreases in both the number and the average cross-sectional area of Aβ plaques. Upregulation of CD68 in plaque-apposed microglia is consistent with the disease-associated microglia phenotype and reflects improved microglial capacity for Aβ clearance in vivo [69, 70]. Notably, the SULF A-driven phagocytic response appeared to be restricted to Aβ plaque niche since we found no evidence of excessive engulfment of synaptic material, supporting a selective Aβ plaques-directed clearance mechanism that preserves synaptic elements.

Notably, this engagement was achieved without signs of neuroinflammation, contrasting sharply with antibody therapies that frequently trigger ARIA and vasogenic oedema, raising concerns about long-term safety in broader populations [65].

Microglia exhibit remarkable plasticity and can adopt a range of phenotypes in response to environmental cues. SULF A appears to fine-tune microglial activation, enhancing functional capabilities such as motility and phagocytosis without triggering classical pro- or anti-inflammatory cytokine release. Indeed, at the cellular level, SULF A enhances the phagocytosis of multiple Aβ species, with a pronounced effect also on soluble oligomers, which are more neurotoxic and resistant to clearance by quiescent microglia [71]. Despite the centrality of Aβ clearance in AD pathology, few studies have investigated the impact of small molecules on microglial phagocytosis of distinct Aβ aggregate species. Using fluorescently labeled Aβ₁_−_₄₀ peptides which faithfully recapitulate the dynamic aggregation behavior of endogenous Aβ, we demonstrated that SULF A significantly increased uptake of monomeric, protofibrillar, and fibrillar aggregates by primary murine microglia. This selective enhancement of oligomeric Aβ uptake is particularly noteworthy given recent discoveries linking these diffusible species to synaptic loss, oxidative stress, and tau pathology [72, 73]. The ability of SULF A to promote oligomer clearance supports the emerging hypothesis that improving microglial function may yield greater therapeutic prospective [67]. Indeed, while microglia are initially recruited to clear Aβ deposits, prolonged activation has been shown to impair their phagocytic capacity, leading to inefficient clearance of neurotoxic aggregates [74]. Strategies aimed at restoring microglial phagocytosis are increasingly recognized as promising approaches for disease-modifying therapies in AD [75].

Mechanistically, SULF A modulates microglial activation toward a surveillance-oriented, homeostatic state, characterized by increased process complexity, reduced soma size, and elevated motility, which are associated to patrolling behavior and response to subtle pathological changes [76]. This phenotype contrasts with the hypertrophic, amoeboid morphology typically seen in chronically activated microglia in AD [77]. Enhanced motility was further confirmed in microglia-neuron co-cultures, suggesting a heightened capacity to detect and respond to neuronal stress. These structural and behavioral changes are consistent with modulation of TREM2 signaling, a key receptor that regulates actin remodeling, phagocytosis, and lipid sensing in microglia, and whose activity is increasingly associated with therapeutic benefit [78].

Importantly, in analogy with our previous report on DCs [19], SULF A appears to fine-tune immune activation without triggering overt inflammation. Unlike classical M1/M2-polarizing stimuli, SULF A did not induce IL-12, TNF-α, IL-4, or IL-10 production while other activation markers, such as its stimulatory and co-stimulatory signals, remained at functional levels. Notably, we observed a selective increase of CCL2, a chemokine associated with tissue surveillance and immune recruitment, without causing systemic immune activation. Also CD40, a pro-inflammatory co-stimulatory molecule, was significantly downregulated, while *Arg*1 expression was upregulated, leading to a robust increase in the ARG1/iNOS ratio, indicative of a reparative, immune-resolving microglial profile [79]. These data are consistent with a microglial *milieu* that favors tissue monitoring and resolution over inflammation and neurotoxicity [80]. Recent genetic studies have underscored the centrality of innate immunity in AD risk, with key microglial genes such as *TREM2*, *CD33*, and *PLCG2* linked to disease susceptibility [81]. In alternative to current antibody-based AD therapies, the response of microglia to SULF A is based on fine-tuning rather than generalized immune activation, thus mirroring the homeostatic effects we have previously reported on DCs [20].

## Conclusions

This study identifies SULF A as a novel and promising small-molecule candidate for the therapeutic intervention in AD. By promoting microglial surveillance, facilitating the selective clearance of neurotoxic Aβ forms, reducing Aβ plaque burden, and preserving vulnerable neuronal populations, the synthetic sulfolipid delineates a distinctive immunomodulatory strategy centered on neuroprotection and targeted microglia modulation. These effects were consistently observed across both pre-plaque and plaque stages of disease progression, thereby supporting the emerging conceptual framework in neuroimmunology and AD pathogenesis that shifts the focus from mere amyloid clearance toward restoration of immune homeostasis.

## Supplementary Information

Supplementary Material 1:  Supplementary Methods: 1. Microwave-assisted solid phase peptide synthesis of fАβ. 2. Preparation of fluorescent Aβ fibrils. 3. Fluorescence labeled fAβ phagocytosis assay. 4. Immunofluorescence analysis. 5. Morphological analysis of primary microglia. 6. Neuron-microglia cocultures. 7. Time lapse imaging.

Supplementary Material 2:  Supplementary Data: Supplementary Figure 1: SULF A did not induce significative cytokine production by primary murine microglia. Supplementary Figure 2: SULF A-treated cells did not exhibit increased uptake of these non-opsonic targets. Supplementary Figure 3: SULF A enhances microglial phagocytosis and protects against Aβ-induced cytotoxicity.  Supplementary Figure 4: SULF A enhances microglial phagocytosis and protects against Aβ-induced cytotoxicity. Supplementary Figure 5: Cells not exposed to either fAβ or SULF A preserved the typical elongated morphology. Supplementary Figure 6: SULF A enhances microglial phagocytosis and protects against Aβ-induced cytotoxicity. Supplementary Figure 7: CD68 levels in microglia cells near and far from Aβ plaques in old Tg2576 PBS and SULF A-treated mice. Supplementary Figure 8: SULF A does not affect VTA neuroinflammation in pre-plaque Tg2576 mice. Supplementary Table 1: List of the primers used in the qPCR analysis.

Supplementary Material 3: Supplementary Movie 1-2. Time lapse images of microglia and neurons untreated co-culture recorded each 20 min for 24 h.

Supplementary Material 4: Supplementary Movie 1-2. Time lapse images of microglia and neurons untreated co-culture recorded each 20 min for 24 h.

Supplementary Material 5: Supplementry Movie 3-4 Time lapse images of microglia and neurons co-culture treated with 10 µg/mL SULF A and recorded each 20 min for 24 h.

Supplementary Material 6: Supplementry Movie 3-4 Time lapse images of microglia and neurons co-culture treated with 10 µg/mL SULF A and recorded each 20 min for 24 h.

## Data Availability

All data generated or analyzed during this study are included in this published article and its supplementary files. Further information is available from the corresponding author upon reasonable request.

## Abbreviations

SULF A: Sulfavant A
TREM2: Triggering Receptor Expressed on Myeloid Cells 2
Aβ: Amyloid-β
fAb: Aβ₁₋₄₀ labeled with 5(6)-carboxyfluorescein
AD: Alzheimer’s Disease
Arg1: Arginase 1
Ccl2: Chemokine (C-C motif) ligand 2
CNS: Central Nervous System
DAMPs and PAMPs: Damage- and Pathogen-Associated Molecular Patterns
DC: Dentritic cell
DCs: Dendritic cells
GAS: Group A Streptococcus
Iba1: Ionized calcium-Binding Adapter molecule 1
IDPs: Intrinsically Disordered Proteins
iNOS: Inducible Nitric Oxide Synthase
FBS: Fetal bovine serum
LDL: Low-density-lipoprotein
MFI: Mean Fluorescence intensity
NO: Nitric oxide
NORT: Novel Object Recognition Test
OF: Open Field
OFT: Open Field Test
PBS: Phosphate buffer saline
PSD95: Post synaptic density 95
TH: Tyrosine Hydroxylase
Tmem119: Transmembrane protein 119
VTA: Ventral Tegmental Area
WT: Wild type
