# Supplementary material for: Microglial clearance, neuroprotection and cognitive recovery via a novel synthetic sulfolipid in Alzheimer’s disease
Source: J Neuroinflammation. 2025 Dec 13;23:20. doi: 10.1186/s12974-025-03634-w (PMC12821313; doi:10.1186/s12974-025-03634-w)
Supplement: Supplementary file 1 — Supplementary Material 1: Supplementary Methods: 1. Microwave-assisted solid phase peptide synthesis of fАβ. 2. Preparation of fluorescent Aβ fibrils. 3. Fluorescence labeled fAβ phagocytosis assay. 4. Immunofluorescence analysis. 5. Morphological analysis of primary microglia. 6. Neuron-microglia cocultures. 7. Time lapse imaging. [file 12974_2025_3634_MOESM1_ESM.docx]

# Supplementary methods

1. Microwave-assisted solid phase peptide synthesis of fАβ

Amyloid β _1-40_ (Aβ) (purity ≥95%) was obtained by Bachem (Bubendorf, Switzerland). Peptide-synthesis grade N,N-dimethylformamide (DMF) and CH3CN (HPLC Grade) were purchased from Sigma-Aldrich. Fmoc-L-protected amino acids and activators N,N′-diisopropylcarbodiimide (DIC), Oxyma pure, HATU (Hexafluorophosphate Azabenzotriazole Tetramethyl Uronium), benzotriazol-1-yloxytripyrrolidinophosphonium hexafluorophosphate (PyBOP), were purchased from Iris Biotech. Fmoc-L-Val Novasyn TGA resin, 5(6)-carboxyfluorescein (5(6)-FAM), Diethyl ether (Et2O), N,N-diisopropiletilammina (DIPEA), tert-butanol (tBuOH), Trifluoacetic acid (TFA) and scavenger for the cleavage of peptides from resin, Triisopropyl silane (TIS), Thioflavin T (ThT) and all other salts were purchased by Sigma-Aldrich (St.Louis, MO, USA).

Aβ labeled with 5(6)-carboxyfluorescein (fAβ) was synthesized and purified in our laboratory with a purity ≥95%. The peptide was synthesized by a fully automated microwave-assisted solid phase peptide synthesis (MW-SPPS), with the peptide synthesizer Liberty Blue (CEM Corporation, Matthews, NC, USA), according to the Fmoc/tBu strategy on a Fmoc-L-Val Novasyn TGA resin (0,23 mmol/g, 220 mg, 100-200 Mesh) in a 0.05 mmol scale. After resin swelling in DMF, Fmoc-amino acids were introduced through the following protocol: 1) Fmoc-deprotection in 20% piperidine in DMF; 2) washes (1×) with DMF; 3) Fmoc-deprotection in 20% piperidine in DMF; 4) washes (4×) with DMF; 5) coupling with Fmoc-amino acid (5 eq, 0.2 M in DMF), Oxyma pure (5 eq, 1M in DMF) and DIC (5 eq, 0.5 M in DMF) prepared in separated bottles; 6) washes (1×) with DMF; 7) coupling with Fmoc-amino acid (5 eq, 0.2 M in DMF), Oxyma pure (5 eq, 1M in DMF) and DIC (5 eq, 0.5 M in DMF) prepared in separated bottles; 8) washes (1×) with DMF. Both deprotection and coupling reactions were performed in a Teflon vessel with microwave energy and nitrogen bubbling. Reaction temperatures were monitored by an internal fiberoptic sensor. The resin was exposed to the microwave-assisted cycle described according to the following parameters:

| **Step** | **Temperature (°C)** | **Power (W)** | **Time (s)** |
| --- | --- | --- | --- |
| **Deprotection** | 25 | 0 | 300 |
| **Conventional Coupling** | 25 | 0 | 3600 |
| **MW Coupling** | 75 | 160 | 15 |
|  | 90 | 30 | 110 |

For labeling Аβ at the N-terminus, the side-chain-protected peptide resin (0.01 mmol) was acylated with 5(6)-FAM (11,3 mg, 3 eq) 2 times at room temperature by HATU (11.4 mg, 3 eq) and DIEA (7 mL, 4 eq) and a third time replacing the HATU with PyBOB (13 mg, 3eq) during overnight. Final cleavage from the resin, with concomitant side chains deprotection was achieved by treatment of resin-bound peptide with a TFA/TIS/H_2_O/EDT solution (92.5:2.5:2.5:2.5), 1 mL mixture/100 mg of resin). The mixture was stirred for approximately 4 h at room temperature. The resin was filtered and then rinsed with TFA (2 × 1 mL). The peptide solution was added to the washes and the product was precipitated from this solution by the addition of ice-cold Et_2_O (30 mL). The precipitated material was washed with ice-cold Et_2_O (3 x 30 mL) and dried under vacuum. The solid peptide was then dissolved in H_2_O (5 mL), iced at -30 °C overnight and lyophilized. The resulting crude peptide fАβ was purified by semi-preparative Reversed Phase HPLC (RP-HPLC) using a fully automated Waters Preparative System equipped with a Phenomenex Synergi 4u Fusion-RP C18 (150 × 10 mm) column operated at 4 ml/min at 25 °C. The solvent systems used were A (0.1% TFA in H_2_O) and B (0.1% TFA in CH3CN).

The product was characterized by analytical RP-UPLC-ESI-MS (Waters Acquity UPLC coupled to a Waters 3100 ESI-SQD MS) supplied with a Luna Omega PS C18 (1.6 μm, 2.1 × 50 mm) column at 35 °C at 0.6 ml/min with solvent systems A (0.1% TFA in H_2_O) and B (0.1% TFA in CH_3_CN) using the following method: gradient 15-40% B in 3.5 min, Rt 2.9. Mass spectra were recorded on a simple Quadrupole 3100 MS detector instrument on Electrospray positive mode. The experimental conditions for spectra acquisition in the positive ion mode were: spray voltage=3.0kV, Cone Voltage=20V, Desolvatation Temperature= 400°C, capillary temperature= 250 °C; m/z range= 200–2000. Data were acquired and processed using MassLynx software (Waters, Milford, MA, USA). ESI-MS [obsd: m/z (M+3H)^3+^ 1563.72; (M+4H)^4+^ 1173.24; calcd: m/z (M+H)^+^ 4689.5.

1. Preparation of fluorescent Aβ fibrils

To obtain fluorescent sample without significantly perturbing the structure of intermediate species of amyloid aggregation process, after some optimization trials, we co-incubated Aβ with a small percentage of fAβ (1.25%) at 37°C under gentle shaking. We monitored the aggregation process through a ThT experiments. Briefly, we added 20 µM of ThT to 100 μL of our sample solution. ThT experiments were carried out in Corning 96 well non-binding surface plates. Time traces were recorded using a Victor Nivo 3S (Perkin Elmer, Waltham, MA) plate reader using a 20 nm bandpass filter at 435 nm for excitation and a 30 nm bandpass filter at 480 nm for emission at 37 °C, shaking the samples for 10 s before each read. Samples (200 μL) were collected at 0, 1, 23 and 48 hrs and frozen it at -80 °C to stop the aggregation process.

3. Fluorescence labeled fAβ phagocytosis assay

Primary murine microglia cells were seeded in 24-well plates (3· 10^5^ cells/well) and incubated with SULF-A (10 μg/mL) and each of 1 µM DFAM beta amyloid (1,40; 1:20) for 1 hour as previously reported. At the end of the treatment, cells were washed three times with PBS, stained with propidium iodide for vitality analysis and acquired by MACSQuant® Analyzer 16 (Miltenyi Biotec). For imaging samples were fixed in 4% (v/v) paraformaldehyde for 20 min at 4°C. Phagocytosis rate was calculated as described above for IgG FITC beads and E. coli particles assay. Phagocytosis data from microscopy analysis were obtained from total cell fluorescence (CTCF) measure by Image J software by the following equation:

*CTCF = Integrated Density – (Area of selected cell x Mean fluorescence of background readings)*

1. Immunofluorescence analysis

Primary murine microglia cells were cultured on poly-D-lysine coverglass in 24-well plates (10^5^ cells/well). Cells were fixed with 4% paraformaldehyde in phosphate-buffered saline (PBS) for 10 min at room temperature. The cells were permeabilized with 0. 1% Triton and blocked in PBS 1X with bovine serum albumin (BSA) 3% at room temperature for one hour. The following primary antibodies were incubated overnight in humid chamber at 4°C: rabbit anti Iba-1 (No. 019-19741; Wako, 1:400), rat anti Trem2 (RMO139-5J46; Abcam ab86491; 1:200); while as secondary antibodies Texas Red-X goat anti-rat (2217029; T6392 Invitrogen; 1:400) and Alexa Fluor 488 goat anti-rabbit (2557379; A11008 Invitrogen; 1:400) were used. Nuclei were counterstained with DAPI (1μg/mL, D1306, Life Technologies). Samples were examined under confocal laser microscope (Zeiss LSM 700 confocal micro-scope system; Carl Zeiss, Gottingen, Germany) using a 40 × or a 63 × oil-immersion objective (1.4 NA). Optical confocal sections (1 μm) were acquired at 1 Airy unit with a resolution of 1024 × 1024 pixels.

For immunofluorescence on mice, animals were anaesthetized (Rompun/Zoletil) and transcardially perfused with phosphate buffer (PB; 0.1 M, pH 7.4) followed by 4% paraformaldehyde in PB. Brains were postfixed in 4% paraformaldehyde for at least 4h, dehydrated and cryoprotected in 30% sucrose in PB at 4 °C until sinking. 30 μm-thick coronal sections were cut with a cryostat, and slices were collected in PB-Sodium Azide 0.02%.

For CD68/6E10/Iba1 staining, brain slices were permeabilized using 0.5% Triton X-100 in PB (45 min, RT), incubated in blocking solution (2% bovine-serum albumin, 0.5% Triton X-100 in PB) containing M.O.M.® (Mouse on Mouse) Blocking Reagent (1:1000; Vector laboratories, #MKB-2213-1), for 2 h at RT, then exposed to primary antibodies in blocking solution (2 nights, 4 °C): anti-CD68 (1:400; Biorad #MCA1957; RRID:AB_322219), anti-hAPP695 (6E10; 1:500, BioLegend #803001; RRID: AB_2564653), anti-Iba1 (1:700; Wako #019-19741; RRID:AB_839504). For PSD95/CD68/Iba1 staining, brain slices were permeabilized using 2% Triton X-100 in PB (2h, RT), incubated in blocking solution (2% bovine-serum albumin, 10% donkey serum, 0.3% Triton X-100 in PB) containing M.O.M.® (Mouse on Mouse) Blocking Reagent (1:1000; Vector laboratories, #MKB-2213-1), for 2 h at RT, then exposed to primary antibodies in blocking solution (2 nights, 4 °C): anti-CD68 (1:400; Biorad #MCA1957; RRID:AB_322219), anti-PSD95 (1:250, Millipore # MAB1598; RRID:AB_11212185), anti-Iba1 (1:700; Wako #019-19741; RRID:AB_839504). For hippocampal TH^+^ fibers, sections were incubated with primary anti-TH antibody (1:500; Millipore #AB152; RRID: AB_390204) using 0.3% Triton X-100 in PB (overnight 4 °C). For Iba1^+^ cell count and Sholl analysis and TH^+^ neuron count, slices were incubated in PB with 0.3% Triton X-100 with primary antibodies. Anti-Iba1 (1:700; Wako #019-19741; RRID: AB_839504), anti-TH (1:1000; Millipore #MAB318; RRID: AB_2201528). After primary antibodies, slices were incubated with secondary antibodies in blocking solutions (2 h, RT): Alexa Fluor-555 donkey anti-rabbit (1:200; #A31572; RRID:AB_162543), Alexa Fluor-488 donkey anti-rabbit (1:200; #A21206; RRID: AB_2535792), Alexa Fluor-488 donkey anti-mouse (1:200; #R37114; RRID:AB_2556542), Alexa Fluor-555 donkey anti-mouse (1:200; #A31570; RRID: AB_2536180), Alexa Fluor-647 goat anti-rat (1:200; #A-21247; RRID:AB_141778). Slices were then counterstained with DAPI (1:1000, Serva), mounted, and examined using a Nikon Eclipse Ti2 confocal microscope using a 20x objective or a 60x oil-immersion objective and acquired as a z-stack. Optical confocal images were taken at high resolution (pixel size 1024x1024) at 1.2 Airy unit. The labelling specificity was confirmed by omission of primary antibodies and use of normal serum instead (negative controls).

For Iba1 cell count around Aβ plaques, microglia cells were classified as “plaque-associated” if their soma centroid was located within 10 μm of an individual cortical Aβ plaque boundary (6E10-labelled), and as “distant” if located beyond this range. Microglia cells were manually counted and the number was normalized on the single Aβ plaque area adapted from^1^. For CD68 analysis, only isolated (non-overlapping) Iba1^+^ cells around and within Aβ plaques were considered. Individual microglial soma was delineated using the Iba1 channel. Z-stack images (z-step 0.5 µm) were processed simultaneously and analyzed with Fiji-ImageJ(http://imagej.nih.gov/ij/): after 8-bit conversion and background subtraction, CD68 level was quantified by measuring the relative fluorescence intensity within a single non-overlapping microglia cellular soma. To analyze the number of Aβ plaques in the cortex, we acquired images with a 10x-objective at high resolution (pixel size 1024x1024) at 1.5 Airy unit. The mean number of 6E10^+^ plaques in the cortex from at least 4 slices/animal was quantified by using NIS-Elements software (Nikon® Instruments Inc.). The Aβ plaque area was quantified with ImageJ on Z-stack images (z-step 0.5 µm) acquired at 60x-oil objective.

For PSD95 synaptic puncta colocalizing with microglial CD68+, z-stack images (z-step 0.5 µm) were acquired at 60x-oil objective and quantification was performed using ImageJ. Briefly, a mask defining the microglia cell area (ROI) was created and measured to determine total cell surface. A second mask was created in CD68 channel after 8-bit conversion and background subtraction (using the rolling ball method). Then PSD95^+^ synaptic puncta were quantified by a semi-automatic counting (Analyze Particle plug-in using a size range of 0–0.5 µm²) within the ROI. Puncta were visually inspected and manually corrected when necessary. For the colocalization, the Image Calculator function was applied using the logical AND operation between the CD68 and PSD95 masks to quantify PSD95-positive puncta located within CD68-positive regions.

For TH^+^ fiber density analysis, images were acquired with a 20x-objective by Z-stacks (z-step 1.1 µm), then processed by maximum-intensity projection. Samples were captured with identical Z-stack thickness and laser settings. 3D-images were collected from at least 4–5 slices processed simultaneously. The fiber density was quantified manually from the different hippocampal subfields (CA1, CA3, Dentate Gyrus; DG) and was expressed as number of fibers/ 250 μm [25]. Fiber density for the total hippocampus was obtained by averaging the density of each subfield per animal.

1. Morphological analysis of primary microglia

Microglia morphology was classified as previously reported ^2^: ramified (reduced cell body with multiple thin and long processes), polarized (reduced cell body with one or two thin processes) and amoeboid (increased cell body with no or few retracted processes). The analysis was performed by manually counting the cell according to their morphology with Cell counter plug-in on ImageJ software (NIH). Experiment was performed in triplicate with at least 20 randomly selected fields for each condition: 796 not treated cells and 754 SULF-A cells were analyzed. Chi-square test was applied **** p<0.0001

1. Neuron-microglia cocultures

Primary cortical neurons were prepared from wild type neonatal brains. Cortices were dissected and dissociated to single cells suspension with 0.125% trypsin. Cells were plated in poly-lysine coated 24 wells (1.5 x 10^5^ cells/well) and maintained in Neurobasal medium (NB, Gibco, Carlsbad, CA, USA), 1% B-27 plus supplement (Gibco, Carlsbad, CA, USA), 10% Horse serum (Gibco, Carlsbad, CA, USA), 2 mM L-glutamine and penicillin/streptomycin (Gibco, Carlsbad, CA, USA). At DIV 4, cells were treated with 10mM Cytosine arabinoside (Sigma-Aldrich, St. Louis, MO, USA) for 48 hours. Half media change was performed every two days. At DIV 11, primary purified microglia was added to neuronal cultures at 1:2 = neurons:microglia ratio.

Mixed glial cells were prepared from P0-P1 neonatal brains of wild type mice (pink arrow), while primary neurons were prepared from cortices of P1 wild type mice (green arrow). After 14-15 days of the glial cells culturing (DIV14-15) primary microglia were detached from the mixed glial cultures by orbital shaking and CD11b+ MACS purification. Microglia cells were plated on DIV11 cortical primary neurons. The following day, 10 µg/mL SULF A treatment was added to neurons-microglia cultures, and cells were observed at time lapse microscopy for 24 hours.

**
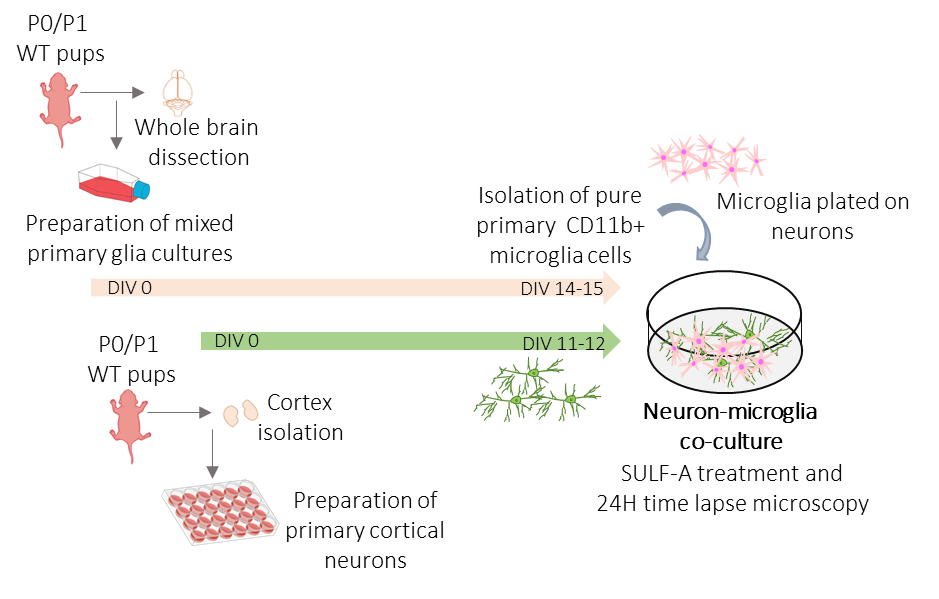
**

1. Time lapse imaging

Time-lapse imaging was performed using a Zeiss inverted motorized Observer Z1 epifluorescence microscope equipped with environment control boxes at 37 °C and a digital camera (CCD) (Carl Zeiss MicroImaging GmbH Jena Germany). Images were acquired in transmission (Phase-contrast) channels with a 40X air objective. Usually, stacks about 6 μm thick, composed of sections separated by 2.0 μm, were taken every 20 minutes during an average period of 24 hours. The captured images thus obtained were mounted using Zeiss ZEN Blue Software (Carl Zeiss Micro Imaging GmbH Jena Germany) and images of one focal plane at the middle-point of a z-stack were used for image analysis.

Reference:

1. Natunen, T. *et al.* Diabetic phenotype in mouse and humans reduces the number of microglia around β-amyloid plaques. *Mol Neurodegener* **15**, 66 (2020).

2. He, Y., Taylor, N., Yao, X. & Bhattacharya, A. Mouse primary microglia respond differently to LPS and poly(I:C) in vitro. *Sci Rep* **11**, 10447 (2021).
