# Supplementary material for: Microglial clearance, neuroprotection and cognitive recovery via a novel synthetic sulfolipid in Alzheimer’s disease
Source: J Neuroinflammation. 2025 Dec 13;23:20. doi: 10.1186/s12974-025-03634-w (PMC12821313; doi:10.1186/s12974-025-03634-w)
Supplement: Supplementary file 2 — Supplementary Material 2: Supplementary Data: Supplementary Figure 1: SULF A did not induce significative cytokine production by primary murine microglia. Supplementary Figure 2: SULF A-treated cells did not exhibit increased uptake of these non-opsonic targets. Supplementary Figure 3: SULF A enhances microglial phagocytosis and protects against Aβ-induced cytotoxicity. Supplementary Figure 4: SULF A enhances microglial phagocytosis and protects against Aβ-induced cytotoxicity. Supplementary Figure 5: Cells not exposed to either fAβ or SULF A preserved the typical elongated morphology. Supplementary Figure 6: SULF A enhances microglial phagocytosis and protects against Aβ-induced cytotoxicity. Supplementary Figure 7: CD68 levels in microglia cells near and far from Aβ plaques in old Tg2576 PBS and SULF A-treated mice. Supplementary Figure 8: SULF A does not affect VTA neuroinflammation in pre-plaque Tg2576 mice. Supplementary Table 1: List of the primers used in the qPCR analysis. [file 12974_2025_3634_MOESM2_ESM.docx]

**Supplementary Figure 1**. SULF A didn’t induce significative cytokine production by primary murine microglia. Cytokine release quantified by ELISA: A) IL12p70, B) IL-4 and C) TNF-α from murine primary microglia not treated (CTRL) and at 48 h of treatment (n = 16) with 10 µg/mL Sulfavant A (SULF A). IL-10 secretion from ELISA assay showed undetectable values from both not treated (CTRL) and SULF A treated microglia samples.


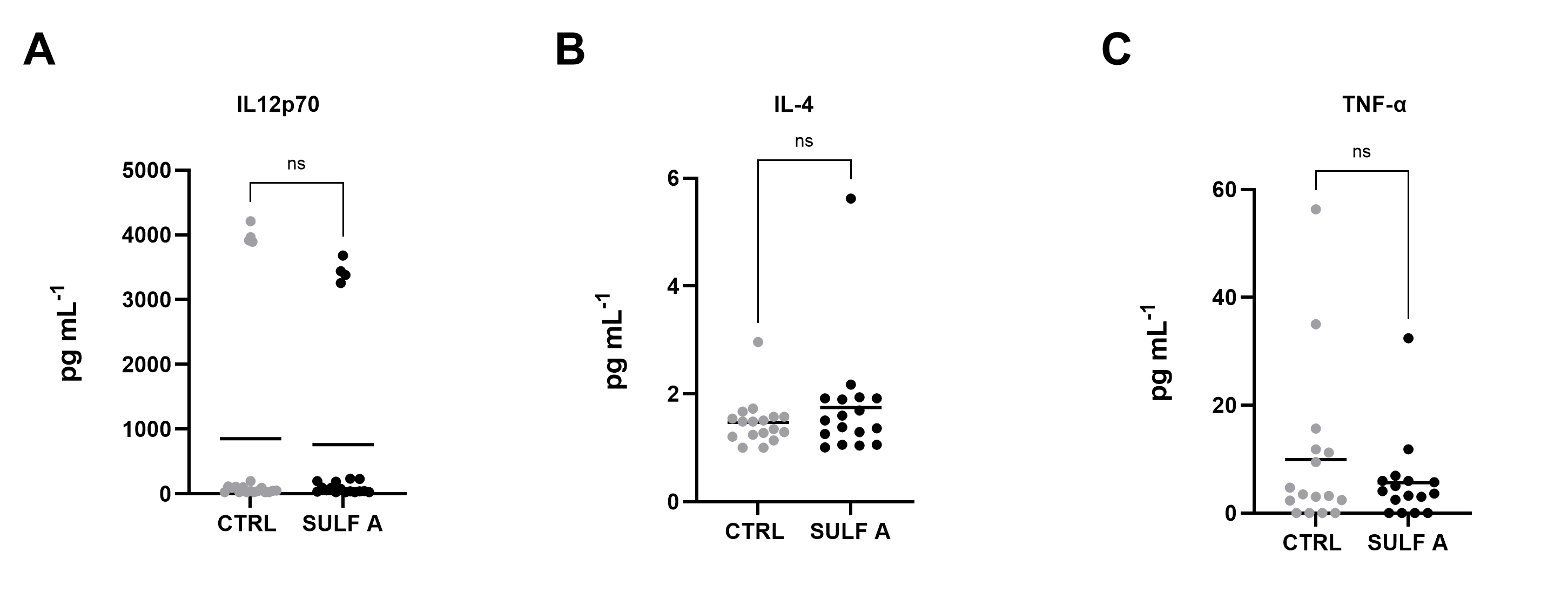


**Supplementary Figure 2**. SULF A-treated cells did not exhibit increased uptake of these non-opsonic targets. Phagocytosis assay conducted on primary microglia untreated (CTRL) and stimulated with SULF A (10 µg/mL) for 3 hours (n = 4) with *E. coli* bioparticles. Data are expressed as Mean Fluorescence Intensity (MFI) quantified by flowcytometry.

**
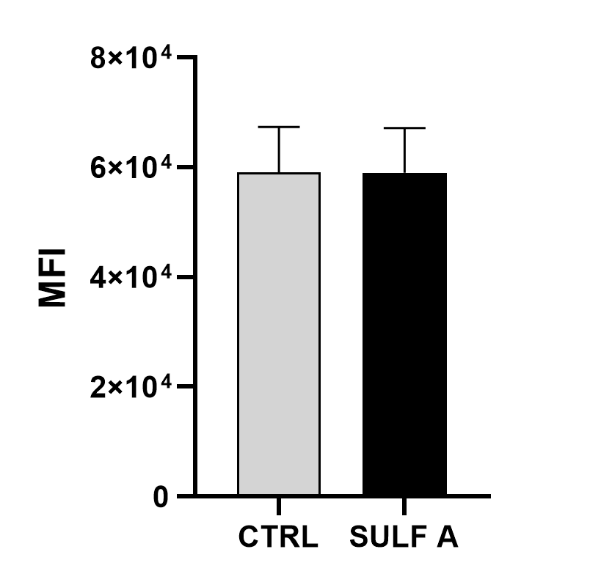
**

**Supplementary Figure 3**. *SULF A enhances microglial phagocytosis and protects against Aβ-induced cytotoxicity*. Representative confocal microscopy images of murine primary microglia stained with DAPI (blue) and Iba1 (red) after phagocytosis assay with SULF A (10 µg/mL) for 3 hours and monomers (1), oligomers (2), prefibers (3) and fibers (4) of fAβ fibrillar fAβ (green). Images were acquired on a Zeiss LSM 700 confocal microscope, with a 63X objective (NA 1.4) and a zoom of 1.5 and 2 respectively.


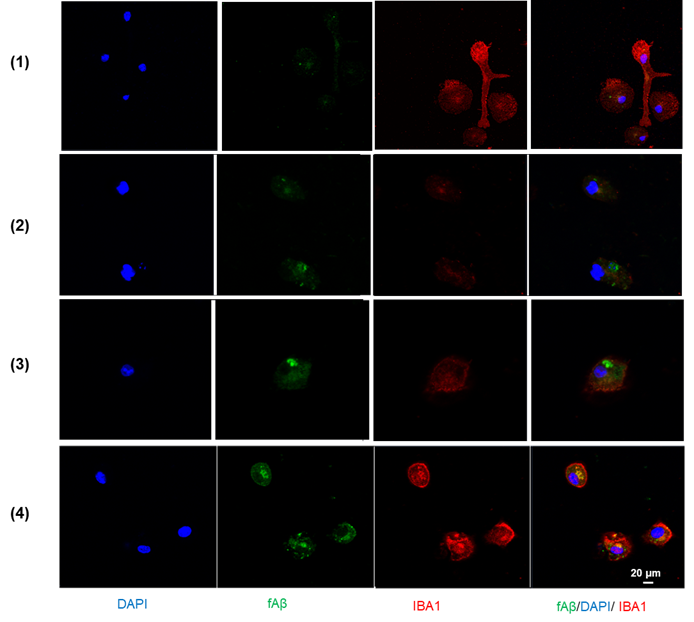


**Supplementary Figure 4.** *SULF A enhances microglial phagocytosis and protects against Aβ-induced cytotoxicity*. Representative confocal microscopy images of untreated murine primary microglia stained with DAPI (blue) and Iba1 (red) after phagocytosis assay for 3 hours with only monomers (1), oligomers (2), prefibers (3) and fibers (4) of fAβ fibrillar fAβ (green). Images were acquired on a Zeiss LSM 700 confocal microscope, with a 63X objective (NA 1.4) and a zoom of 1.5 and 2 respectively.


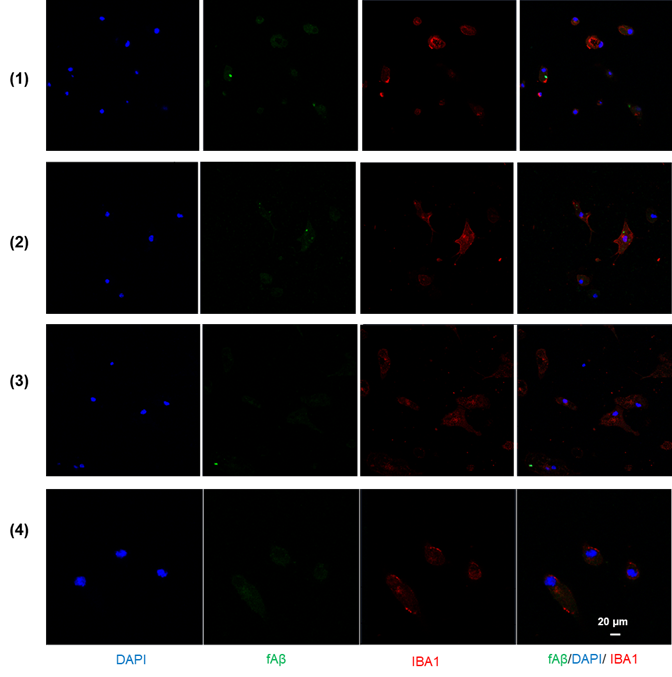


**Supplementary Figure 5.** Cells not exposed to either fAβ or SULF A preserved the typical elongated morphology. Representative confocal microscopy images of untreated murine primary microglia stained with DAPI (blue) and Iba1 (red) Images were acquired on a Zeiss LSM 700 confocal microscope, with a 40X objective.


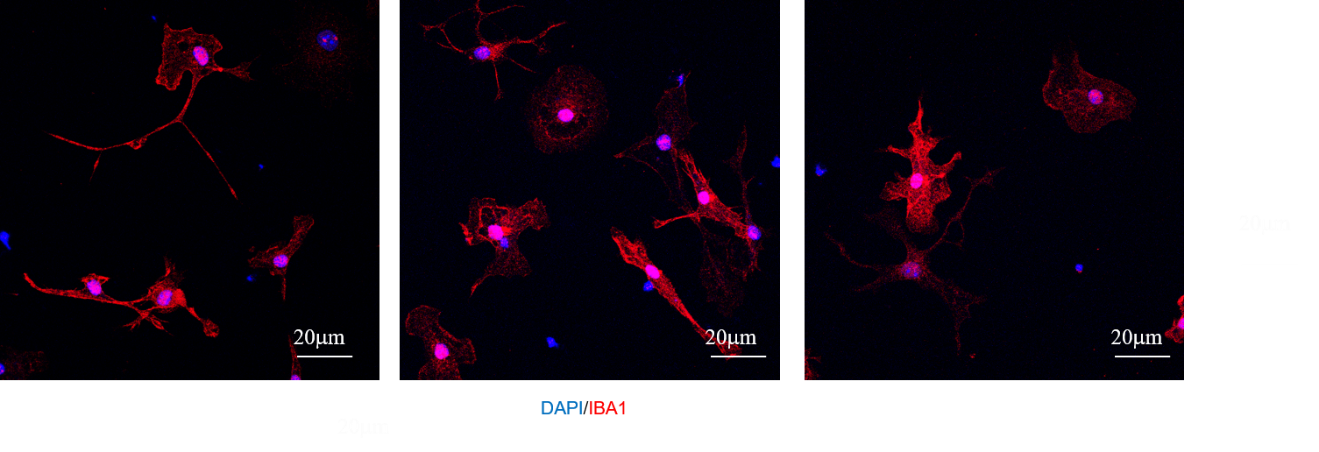


**Supplementary Figure 6.** *SULF A enhances microglial phagocytosis and protects against Aβ-induced cytotoxicity***.** Representative confocal microscopy images of A) fAβ and B) fAβ + SULF A treated murine primary microglia stained with DAPI (blue) and Iba1 (red) after phagocytosis assay for 3 hours with fAβ (green). Images were acquired on a Zeiss LSM 700 confocal microscope, with a 63X objective (left) and 100X objective (right).

**
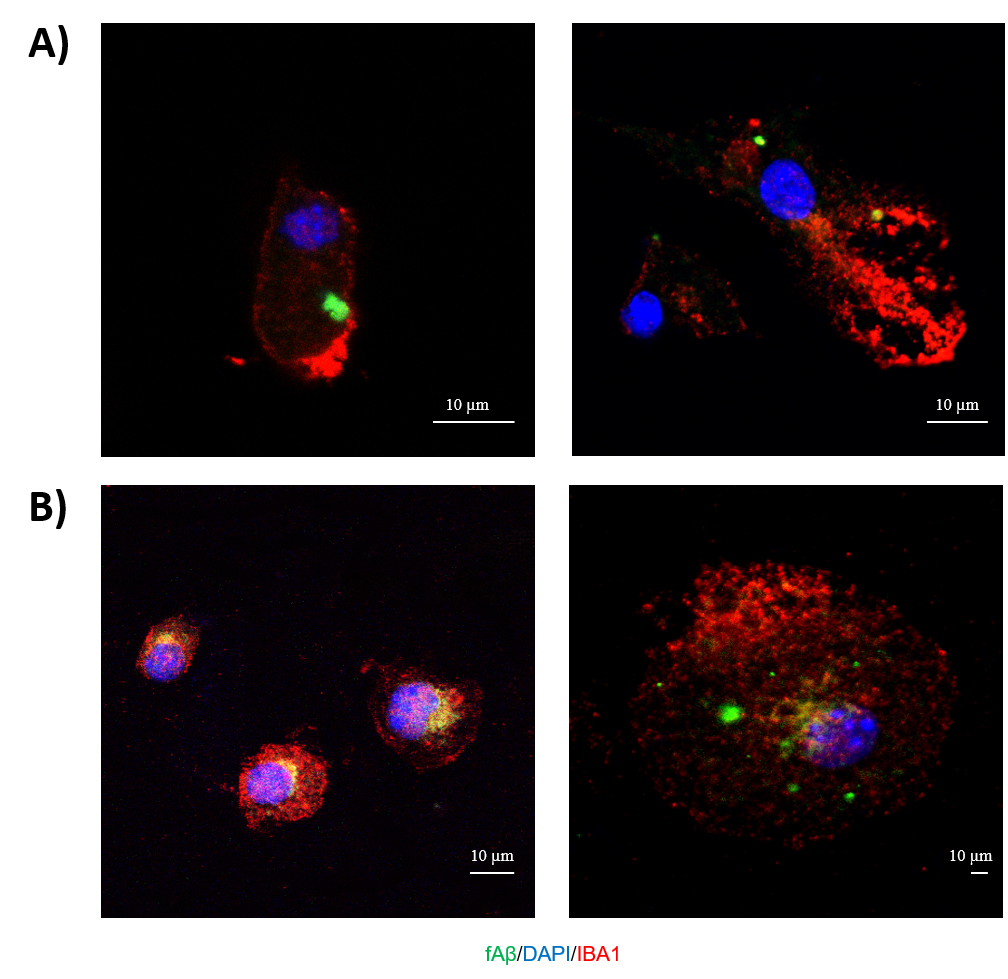
**

**Supplementary Figure 7:** *CD68 levels in microglia cells near and far from Aβ plaques in old Tg2576 PBS and SULF A-treated mice*. A) Representative confocal images of CD68 levels (green) in microglia cells (red; scale bar: 2 μm) in Tg PBS *vs* Tg SULF A near (*top panel*; within 10 μm) and far (*bottom panel*; > 10 μm) from cortical Aβ plaques. The plot shows the somatic levels of CD68 in Iba1^+^ cells (*near Aβ plaque* Tg PBS: n= 21 cells; Tg SULF A n = 23 cells; *far from Aβ plaque* Tg PBS: n=26 cells; Tg SULF A: n=25 cells; n = 4 mice per group; Two-way ANOVA: interaction F_1, 91_= 5.992, **P* = 0.0163; treatment F_1, 91_= 10.54 ***P* = 0.0016; distance F_1, 91_ = 114.8 p < 0.0001; Tg PBS close Aβ *vs* Tg SULF A close Aβ ***P* = 0.0011; Tg PBS close Aβ *vs* Tg PBS far Aβ *****P* < 0.0001; Tg SULF Aβ close *vs* Tg SULF A far Aβ *****P* < 0.0001 analyzed with Tukey's multiple comparisons test). B) Additional information about microglial synaptic engulfment: split channels of PSD95^+^ puncta (red) colocalizing with CD68^+^ lysosomes (cyan) and merged channels (scale bar: 2 μm).

**
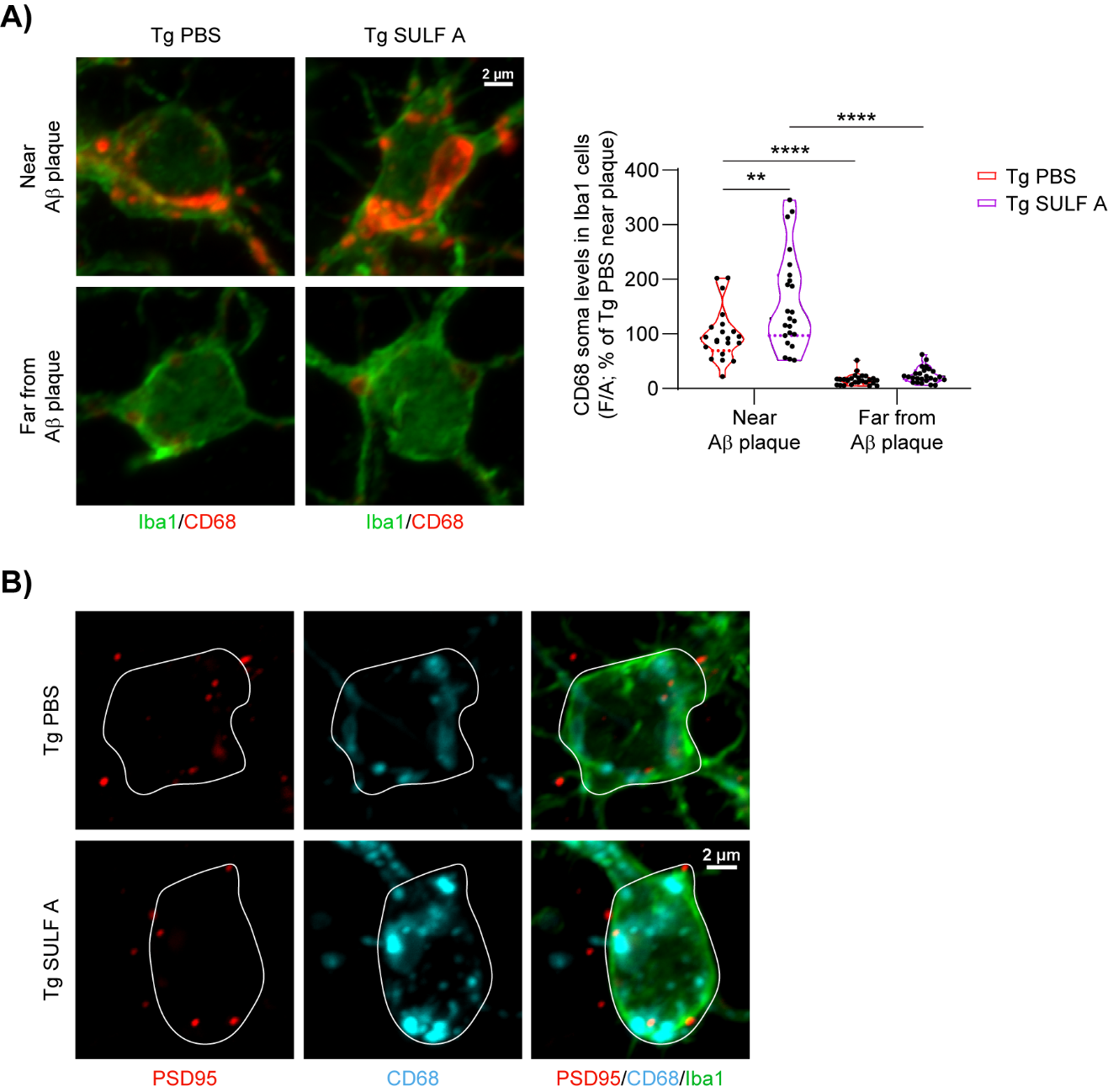
**

**Supplementary Figure 8**. *SULF A does not affect VTA neuroinflammation in pre-plaque Tg2576 mice.* A) Representative confocal images of Iba1^+^ cell (red; scale: 50 μm) and plot showing VTA Iba1^+^ cell count in 6-month-old PBS- and SULF A-treated Tg2576 mice (n = 4 mice/group). Nuclei are counterstained with DAPI. B) Representative confocal images and 3D-reconstruction of microglia (red; scale bar: 10 μm). The graphs show microglia Sholl analysis, depicting number of intersections, nodes, endings and dendritic length at radial distances from the soma (n = 4 mice/group; RM-ANOVA: *Intersections*: Interaction F_6, 36_ = 0.2517, p = 0.9554, radius F_6, 36_ = 408.3, *P* < 0.0001, treatment F_1, 6_ = 2.538 *P* = 0.1622; *Nodes*: Interaction F_6, 36_ = 0.2555, *P* = 0.9538, radius F_6, 36_= 195.1, p<0.0001, treatment F_1, 6_ = 0.1295, p=0.7313; *Endings*: Interaction F_6, 36_=0.2321, *P* = 0.9633, radius F_6, 36_= 145.2, *P* < 0.0001, treatment F_1, 6_ = 0.4408, *P* = 0.5336; *Ramification length*: Interaction F_6, 36_ = 0.2112, *P* = 0.9709, radius F_6, 36_ = 420.3, *P* < 0.0001, treatment F_1, 6_ = 0.7041, *P* = 0.4336) nd somatic perimeter and area (n = 4 mice/group).


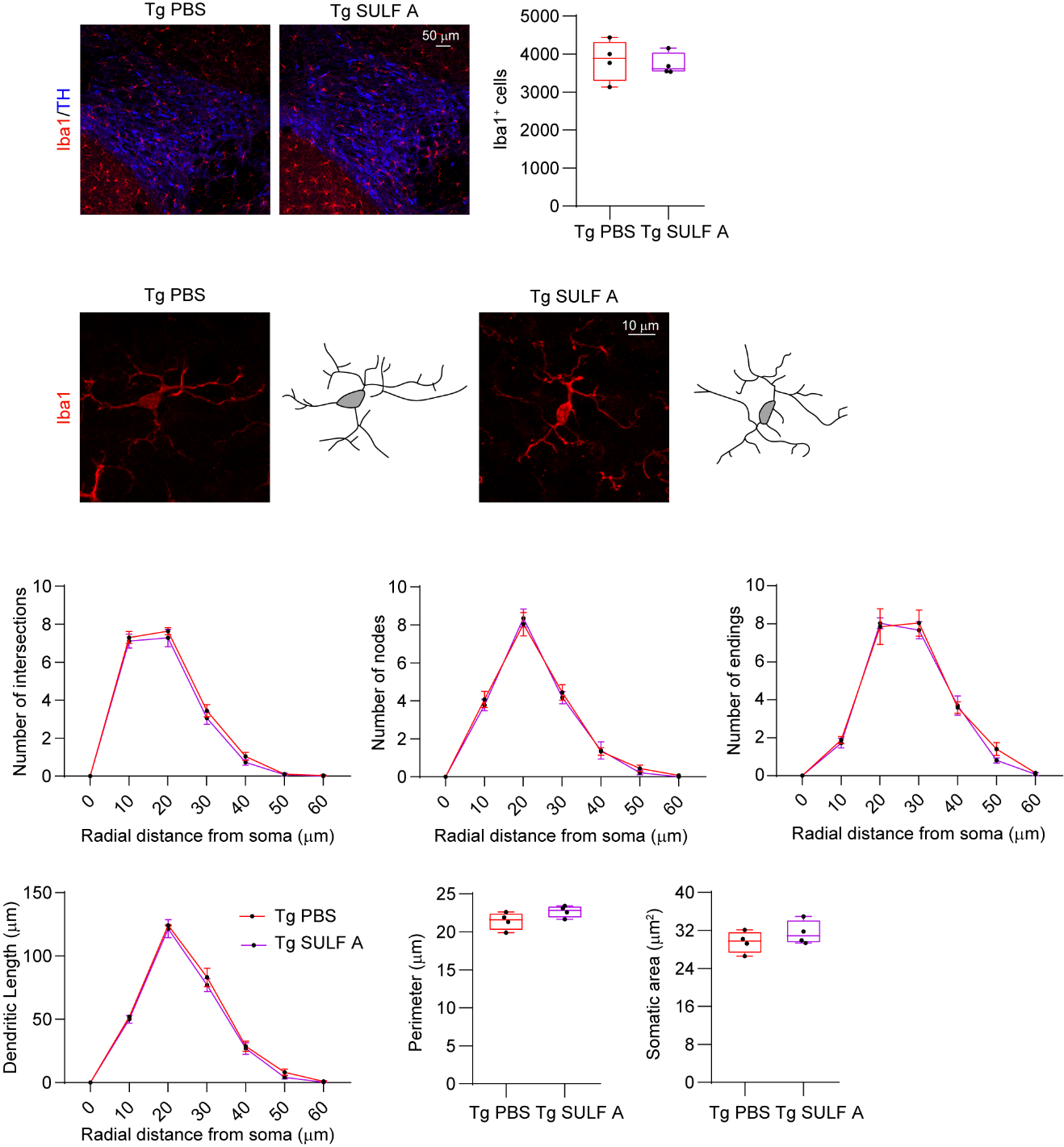


**Supplementary Table 1**. All the primers used in the qPCR analysis.

| **Gene** | **Forward primer (5’-3’)** | **Reverse primer (3’-5’)** |
| --- | --- | --- |
| 18S | GTAACCCGTTGAACCCCATT | CCATCCAATCGGTAGTAGCG |
| *Trem2* | TGGTCAGAGGGGCTGGACTGT | TCCTGGCTGGACTTAAGCTGTAG |
| *Arg1* | GCAGCAGCCGCTGGAACCCAG | GTCCCCGTGGTCTCTCACGTC |
| *Iba1* | GCAGGAAGAGAGGCTGGAGGGGATC | CTCTTCAGCTCTAGGTGGGTCTTCGG |
| *Tnfα* | GCCTCTTCTCATTCCTGCTTGTGGCAG | GACGTGGCCTACAGGCTTGTCACTCG |
| *Tmem119* | TGCAATGTCGCTGTCACTCT | AGTTTGTGTTTCCACGGGGT |
| *Ccl2* | CACTCACCTGCTGCTACTCATTC | GCTTCTTTGGGACACCTGCTG |
| *IL12-p40* | CTCATGGCTGGTGCAAAGAA | CATTCCACATGTCACTGCCC |
| *IL-10* | CATGGCCCAGAAATCAAGGA | GGAGAAATCGATGACAGCGC |
| *IL-4* | CCACGGATGCGACAAAAATC | CACATCCATCTCCGTGCATG |
